# Diet–Microbiota Interactions Alter Mosquito Development

**DOI:** 10.3389/fmicb.2021.650743

**Published:** 2021-06-08

**Authors:** Vincent G. Martinson, Michael R. Strand

**Affiliations:** ^1^Department of Entomology, University of Georgia, Athens, GA, United States; ^2^Department of Biology, University of New Mexico, Albuquerque, NM, United States

**Keywords:** *Aedes aegypti*, microbiota diversity, host–microbiota, microbe–microbe, diet–microbe

## Abstract

Gut microbes and diet can both strongly affect the biology of multicellular animals, but it is often difficult to disentangle microbiota–diet interactions due to the complex microbial communities many animals harbor and the nutritionally variable diets they consume. While theoretical and empirical studies indicate that greater microbiota diversity is beneficial for many animal hosts, there have been few tests performed in aquatic invertebrates. Most mosquito species are aquatic detritivores during their juvenile stages that harbor variable microbiotas and consume diets that range from nutrient rich to nutrient poor. In this study, we produced a gnotobiotic model that allowed us to examine how interactions between specific gut microbes and diets affect the fitness of *Aedes aegypti*, the yellow fever mosquito. Using a simplified seven-member community of bacteria (ALL7) and various laboratory and natural mosquito diets, we allowed larval mosquitoes to develop under different microbial and dietary conditions and measured the resulting time to adulthood and adult size. Larvae inoculated with the ALL7 or a more complex community developed similarly when fed nutrient-rich rat chow or fish food laboratory diets, whereas larvae inoculated with individual bacterial members of the ALL7 community exhibited few differences in development when fed a rat chow diet but exhibited large differences in performance when fed a fish food diet. In contrast, the ALL7 community largely failed to support the growth of larvae fed field-collected detritus diets unless supplemented with additional protein or yeast. Collectively, our results indicate that mosquito development and fitness are strongly contingent on both diet and microbial community composition.

## Introduction

The digestive tract of multicellular animals is the location of nutrient acquisition and absorption, while it is also an ecosystem that hosts communities of microorganisms that are capable of altering animal metabolism, physiology, and development (Sommer and Bäckhed, 2013; Moran et al., 2019). These gut-associated microbial communities can modify animal nutrition by directly serving as a food source or producing factors that have nutritive, digestive, or signaling functions (e.g., vitamins not found in the diet, digestion of inaccessible compounds in the diet) (Dadd, 1973; Claesson et al., 2012; Russell et al., 2013; Wong et al., 2014; Yamada et al., 2015; Singh et al., 2017; Bing et al., 2018). Animals often consume diets that vary in macronutrient composition that affects the overall nutritional content and the gut microbiota can vary in species membership, community complexity, and abundance (Moeller and Ochman, 2013; Martinson et al., 2017; Fast et al., 2018; Youngblut et al., 2019). Interactions between gut microbes and diet can affect a range of physiological processes in both vertebrates and invertebrates; however, it is often difficult to discern how hosts are affected by microbe–microbe vs. microbe–diet interactions because of the complex microbial communities that many animals harbor and variable diets they consume (Ezenwa et al., 2012; Engel and Moran, 2013; McFall-Ngai et al., 2013; Kohl et al., 2014; Wong et al., 2014; Martino et al., 2018; Guilhot et al., 2019; Kovatcheva-Datchary et al., 2019; Zimmermann et al., 2019). Thus, species that are amenable to simplifying and manipulating microbiota composition while controlling diet can help advance understanding of how microbes and diet interact to affect host fitness (Virk et al., 2016; Keebaugh et al., 2018).

In the case of insects, some species feed on highly specialized diets while others including many detritivores consume variable diets that range from nutrient rich to nutrient poor (Douglas, 1998). Most insect detritivores also host communities of microbes in their gut that are acquired from the environment by feeding (Engel and Moran, 2013). Mosquitoes (family Culicidae) are a diverse group (∼3,500 species) of insects that are primarily aquatic detritivores during their juvenile stages while feeding on vertebrate blood (females) and/or carbohydrates (males and females) as adults (Clements, 1992). Blood feeding by adult females can also result in the transmission of pathogens. Mosquitoes host environmentally acquired gut microbiotas that consist primarily of bacteria, but can also include fungi, algae, protozoa, and viruses (Strand, 2018). Microbiota community composition varies greatly within and between mosquito species as a function of collection site and date, life stage, and sex; all factors that alter the microbes that are encountered by individuals (e.g., females encounter blood, while males do not) (Boissiere et al., 2012; Osei-Poku et al., 2012; Yee et al., 2012; Duguma et al., 2013; Minard et al., 2013; Gimonneau et al., 2014; Kim et al., 2015; Yadav et al., 2015; Buck et al., 2016; Coon et al., 2016b; Muturi et al.,2016a,b; Dickson et al., 2017; Thongsripong et al., 2018; Villegas et al., 2018; Malassigné et al., 2020). Regardless of the highly variable microbial communities observed across mosquitoes and similar to many other insects, the species richness of an individual mosquito’s microbiota is much lower than in mammals, which makes them tractable organisms to study how differences in microbial diversity can affect development and nutrition (Yee et al., 2012; Strand, 2018).

*Aedes aegypti* is a mosquito that prefers subtropical–tropical, urban habitats where it blood feeds on humans and can vector the viruses that cause yellow fever, Dengue fever, and Zika syndrome (Kyle and Harris, 2008; Enserink, 2015). Adult females preferentially lay eggs in small, water-holding containers where larvae develop under physical conditions that include non-freezing temperatures and seasonal photoperiods (Service, 1995; Washburn, 1995; Vezzani, 2007). In the laboratory, *A. aegypti* and other mosquitoes are reared by feeding larvae nutrient-rich diets that also support the growth of microbial communities in the aquatic environment (Bond et al., 2017). While laboratory diets differ widely among research groups, a diet consisting of equal parts (w/w) rat chow, heat-killed torula yeast (*Cyberlindnera jadinii*), and lactalbumin (hereafter named RCM diet) has been used to rear *A. aegypti* and several other mosquito species (and aquatic microorganisms) at the University of Georgia Entomology Department since the 1970s (Foster and Lea, 1975). In contrast, the plant-based detritus diets larvae consume in the field contain much lower amounts of protein and other macronutrients such as fats, but also support microbial communities (Merritt et al., 1992; Kaufman et al., 2002; Xu et al., 2008; Wang et al., 2018).

Studies of detritivorous *Drosophila* spp. indicate that the gut microbiota is either non-essential or only minimally affects the growth of larvae fed protein-rich laboratory diets, whereas consumed microbes benefit larvae fed protein-poor diets by serving concurrently as a protein source and promoting signaling activities that regulate growth functions (Shin et al., 2011; Storelli et al., 2011; Yamada et al., 2015; Bing et al., 2018; Keebaugh et al., 2018). Experimental manipulation of the microbiota indicates that *A. aegypti* larvae do not develop beyond the first instar when reared axenically (germ-free), even when fed the nutrient-rich RCM diet (Coon et al., 2014). However, the addition of an individual bacterial species to generate monoxenic, gnotobiotic larvae generally leads to the rapid develop into adults (Coon et al., 2014). Restoration of development is not restricted to a particular species or community of bacteria, but bacteria must be viable (Coon et al., 2014). Addition of living bacteria also activates several signaling pathways in larvae with functions in nutrient sensing and development (Coon et al., 2017; Vogel et al., 2017; Valzania et al.,2018a,b). Altogether, these findings suggest *A. aegypti* requires a gut microbiota when fed the RCM diet and the nutrient-rich composition of this diet suggests this benefit is not due to bacteria serving as a source of protein but instead viable microbes produce factors that dead microbes cannot provide. In contrast to these results, it was shown recently that autoclaved *E. coli* can promote larval mosquito growth in axenic conditions when fed at very high concentrations in combination with additional high-nutrient dietary components; however, development is delayed and fecundity is reduced relative to mosquitoes conventionally reared (Correa et al., 2018).

To further define the community of microbes that *A. aegypti* larvae require when fed RCM diet, we developed a simplified bacterial community that we could manipulate. We also assessed whether microbes that promote growth when larvae are fed RCM diet also similarly do so when fed (1) commercially prepared tropical fish food (FF), which can also be used to rear *A. aegypti* and other mosquito species in the laboratory, or (2) detritus diets that mimicked diets encountered in the field. Our results indicated that microbiota composition minimally affected larvae fed RCM diet, strongly affected larvae fed FF, and largely failed to support development when larvae were fed detritus diets unless supplemented with protein or yeast. Yet strikingly, bacteria grew to comparable abundances in cultures across all diets we tested. Our results overall identify a range of fitness outcomes for *A. aegypti* that depend on both microbiota composition and diet and provide a framework for future studies to identify the microbial-derived components that promote mosquito development.

## Results

### A Simplified Community of Bacteria Produces Progeny of Comparable or Superior Quality With Conventional Rearing

We assembled a microbiota (ALL7) composed of seven taxonomically diverse species of bacteria (Supplementary Table 1) that had previously been identified as gut community members in the field or laboratory populations (Coon et al., 2014, 2016b). Each of these species could also be distinguished from the others by colony morphology, differences in resistance to particular antibiotics, or other visual traits (Supplementary Table 1). RCM and FF diets differ in terms of specific ingredients but contained near identical amounts of protein, fat, and fiber (Supplementary Table 2). Culture flasks containing sterile water were thus inoculated with axenic first instars, RCM or FF diet that had been sterilized by gamma irradiation, and either no microbiota, an ALL7 microbiota, or a conventional microbiota that was collected in March 2018 from a rearing pan containing fourth instars from our laboratory culture of *A. aegypti*. Previous studies indicated that the conventional microbiota in our laboratory culture contained ∼200 species of bacteria (Coon et al., 2014). Axenic larvae fed FF diet with no microbiota under standard rearing conditions (see “Materials and Methods”) remained first instars and died after several days without ever molting, which was identical to what occurs when axenic larvae are fed RCM diet (Coon et al., 2014). Using 1/10 diluted 869 agar (Eevers et al., 2015) to estimate the abundance of bacteria, results indicated that the ALL7 and conventional microbiota grew to comparable densities (10^9^ cfu/ml) in cultures containing RCM or FF diet regardless of whether mosquito larvae were present or absent (Supplementary Figure 1A). Two measures of mosquito fitness, development time to pupation and adult size as estimated by wing length, were also equivalent or superior for progeny in cultures inoculated with the ALL7 microbiota and either RCM or FF diet when compared with cultures inoculated with a conventional microbiota (Supplementary Figure 1B). No significant differences were detected in the adult size of males or females or in the development time to pupa of mosquitoes reared 6 months later using the ALL7 microbiota (Supplementary Figure 1C). In contrast, *A. aegypti* reared with a conventional microbiota collected in September 2018 exhibited small but significant differences in development time and size when compared with the conventional microbiota collected in March 2018, which potentially reflected changes in community composition (Supplementary Figure 1C). Overall, these results indicated that ALL7 is a simplified microbial community that functionally recapitulates or outperforms the microbiota from our conventionally reared culture on two laboratory diets.

### Monoxenic Rearing on FF Diet Adversely Affects Mosquito Fitness

We determined that each bacterium, when cultured individually in flasks with axenic larvae and RCM or FF diet, grew to densities (Supplementary Figure 2A) that were comparable with the estimated density of total bacteria present in cultures inoculated with the ALL7 community (Supplementary Figure 1A). Although each bacterial species alone generally grew to similar densities in cultures containing RCM and FF diet, *Acinetobacter* grew to about 10-fold lower abundance in RCM (Supplementary Figure 2A). Using FF diet further indicated that each species grew to similar densities in both the presence and absence of larvae (Supplementary Figure 2B). We therefore asked if larvae inoculated with individual bacterial species could develop into adults when fed RCM or FF diet.

**FIGURE 1:**
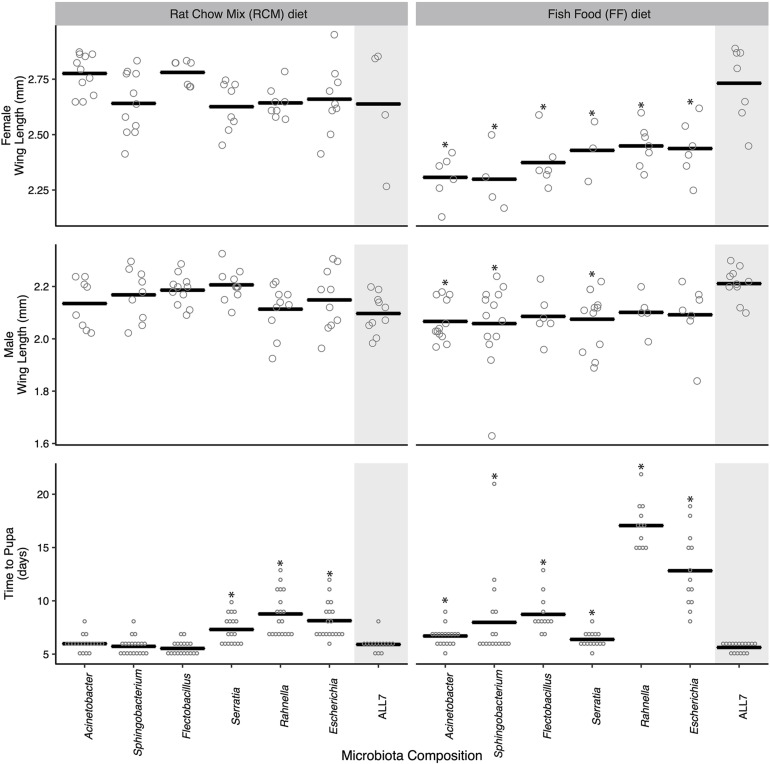
Mosquito development time and adult size when reared monoxenically with different microbiotas on RCM or FF diet. Bars indicate mean abundance for the treatment. Asterisks indicate a significant difference for treatment relative to the community microbiota control = ALL7 [wing length, Dunnett’s (*p* < 0.05); pupation time, Steel’s (*p* < 0.05)].

There were developmental changes in larvae grown with different bacterial species or grown on different diets (Figure 1). No larvae grew beyond the first instar in cultures inoculated with only *Microbacterium*, which similarly occurred in an earlier study where larvae were fed RCM diet and correlated with *Microbacterium* being unable to persist in the larval gut in the absence of other community members (Coon et al., 2014, 2016a). For the other six species in the ALL7 community, monoxenic rearing showed that each supported larval growth to the adult stage (Figure 1). When fed RCM diet, no differences in adult size were detected between these monoxenic treatments and the ALL7 control, but development time to pupation was longer in cultures inoculated with *Serratia*, *Rahnella*, or *Escherichia* (Figure 1). When fed FF diet, development time to pupation was longer than the ALL7 control in cultures individually inoculated with each bacterial species (Figure 1). Cultures inoculated with *Rahnella* or *Escherichia* exhibited especially long delays ($\bar{x}$ = 17.1 and 12.8 days, respectively). Adult females from all monoxenic cultures fed FF diet were also significantly smaller than females from ALL7 control while males in some monoxenic cultures (*Acinetobacter*, *Sphingobacterium*, *Serratia*) were also significantly smaller (Figure 1).

For the preceding assays, the individual mosquitoes within each treatment served as the unit of replication when comparing performance metrics between treatments. However, for a subset of these treatments, we also compared progeny from different culture flasks to assess whether outcomes were consistent with the results presented in Figure 1. Larvae inoculated with the ALL7 community, which exhibited rapid development times and large average adult sizes when fed either RCM or FF diet, exhibited similar developmental times and sizes when progeny from different flasks were compared with one another (Supplementary Figure 3). Further, larvae inoculated with only *Rahnella* or *Escherichia* and fed FF diet exhibited among the longest development times in Figure 1, while comparing progeny from different flasks also showed that developmental rates and adult sizes were similar to one another (Supplementary Figure 3). Thus, development times and adult sizes were generally consistent within each of the aforementioned treatments, while our between-treatment comparisons overall suggested that species composition of the microbial community affected *A. aegypti* development more when larvae were fed FF diet than when fed RCM diet.

### Certain Two-Member Microbiotas Produce Mosquitoes of Similar Quality to the ALL7 Community

**FIGURE 2:**
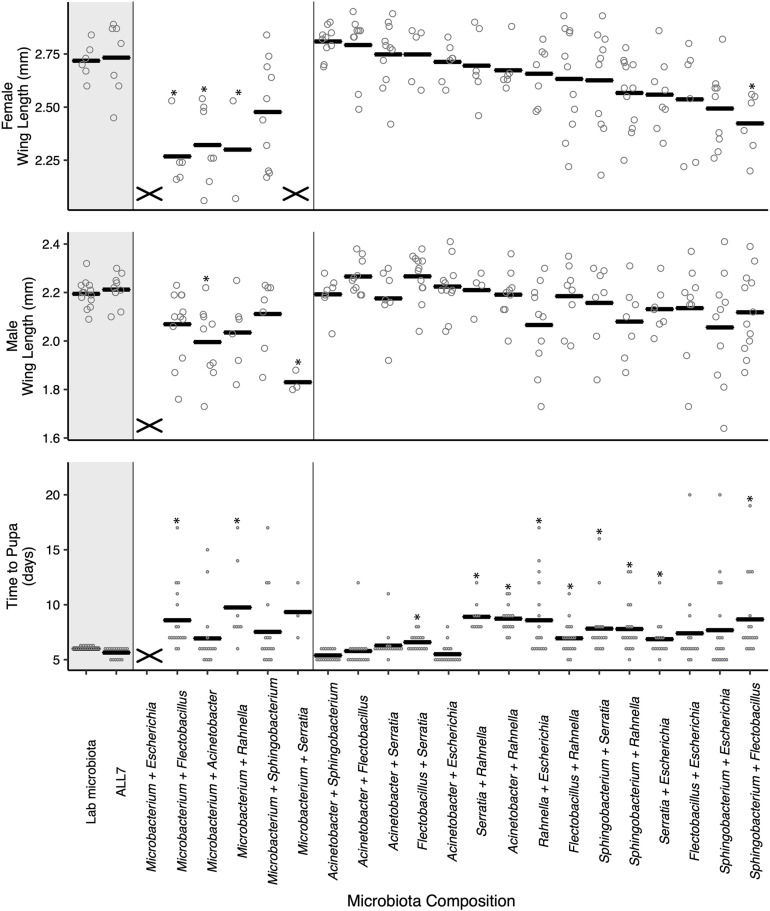
Paired microbial communities largely restore mosquito performance to that of individuals reared with the ALL7 or conventional microbiotas on FF diet. Two-member communities containing *Microbacterium* produced mosquito phenotypes more similar to those monoxenically reared. The “X” marks at the bottom of data columns for “Time to Pupa” and “Wing Length” indicate that larvae did not develop into pupae or adults, respectively. Bars indicate mean abundance for the treatment and asterisks indicate a significant difference for treatment relative to the ALL7 control as in Figure 1.

To further study the outcome of microbial community on *A. aegypti* development, we fed axenic first instars the FF diet and inoculated cultures with the 21 possible pairwise combinations of bacteria from the ALL7 microbiota. Pairwise cultures grew to comparable densities as the ALL7 or monoxenic cultures (Supplementary Figure 4). However, unlike cultures inoculated with only one bacterial species, certain pairwise combinations resulted in development times and adult sizes that did not differ from cultures inoculated with the ALL7 community (Figure 2). Many pairs that included *Microbacterium* had densities at or above that found in the ALL7, yet these microbial communities often resulted in smaller adults and delayed development (Figure 2), indicating that microbial density alone does not explain differences in mosquito development. Pairs that included *Acinetobacter* exhibited development times and adult sizes that were most similar to the ALL7 treatment, while pairs that included *Rahnella* or *Sphingobacterium* exhibited delayed pupation times although delays were shorter than those observed in monoxenic rearing with *Rahnella* or *Escherichia* (Figure 2).

For within treatment comparisons, we selected *Acinetobacter–Escherichia* and *Acinetobacter–Flectobacillus* as examples of two-member communities that performed similarly to the ALL7 community and *Acinetobacter–Rahnella* as an example of a two-member community that exhibited longer development times than the ALL7 community. For each of these two-member communities, development times, and adult sizes were similar when progeny from different flasks were compared with one another (Supplementary Figure 3), which overall provided further support that certain two-member microbial communities promoted development of larvae better than others.

### Manipulating Microbial Community Composition Over Time Affects Mosquito Fitness

Since *Acinetobacter* showed evidence of promoting larval growth in two-member communities while *Rahnella*, *Escherichia*, and *Sphingobacterium* showed evidence of slowing larval growth, we assessed whether manipulating the abundance of these bacteria at different times during development affected *A. aegypti*. This was approached in one set of experiments by inoculating cultures with axenic first instars, FF diet, and either *Escherichia* or *Rahnella* and then adding *Acinetobacter* at the same time (time 0), day 2 post-inoculation, or day 4 post-inoculation (Supplementary Figure 5A). In a second set of experiments, cultures containing axenic larvae and FF diet were inoculated with *Acinetobacter* and either *Sphingobacterium* or *Flectobacillus* at time 0 followed by addition of kanamycin at time 0, day 2, or day 4, which selectively affected *Acinetobacter* (Supplementary Figure 5A). Together, these approaches allowed us to either increase or decrease community diversity in cultures at particular times during larval growth.

**FIGURE 3:**
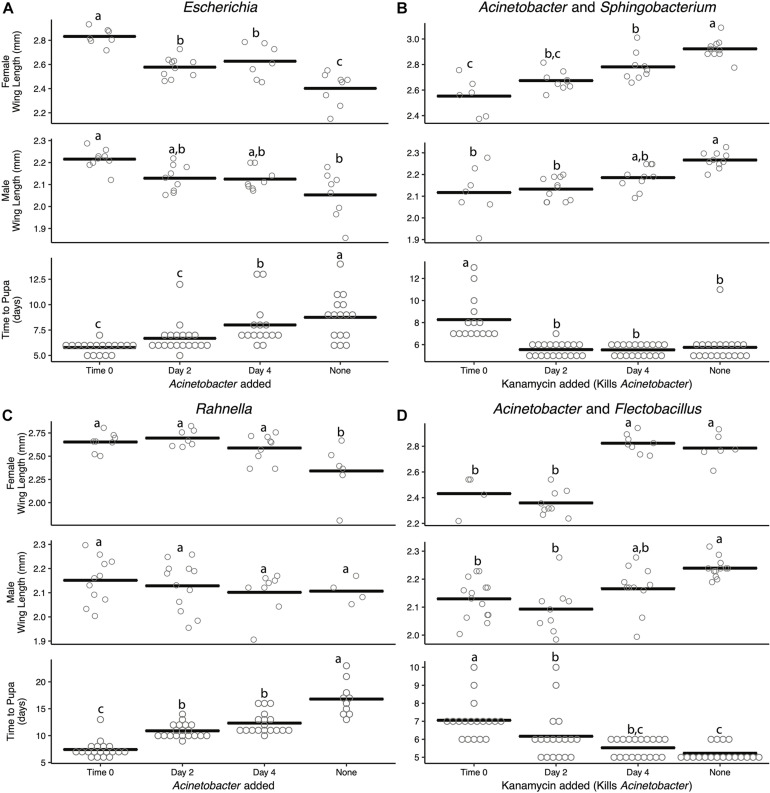
Addition or removal of a second bacterial species during development alters adult size and time to pupa. *Acinetobacter* was added to **(A)** *Escherichia* or **(C)** *Rahnella* or removed with kanamycin from **(B)** *Sphingobacterium* or **(D)** *Flectobacillus*. All experiments were conducted using FF diet. Bars indicate mean abundance for the treatment and significance level assigned by ANOVA with Tukey’s HSD for wing length and Steel–Dwass test for pupation time.

Density estimates showed that adding or removing a second bacterial species resulted in similar colony-forming unit abundances (Supplementary Figure 5B) as found for monoxenic and paired cultures (see Supplementary Figures 2A, 4). Introducing *Acinetobacter* into a monoxenic culture also resulted in it reaching a stable titer within 1 day post-inoculation (Supplementary Figure 5B). The earlier *Acinetobacter* was added to cultures containing only *Escherichia* or *Rahnella*, the greater its effect on reducing larval development times and increasing adult female size (Figures 3A,B). For example, adding *Acinetobacter* to a culture containing *Rahnella* at time 0 decreased mean development time by 44% (16.8 ± 1.01–7.4 ± 0.36 days), while adding at day 2 or 4 reduced development time by 35 and 27% (10.89 ± 0.3 days, 12.33 ± 0.46 days).

Kanamycin treatment of two-member communities resulted in complete elimination of *Acinetobacter* when paired with *Sphingobacterium*, but only partially reduced *Acinetobacter* from a density of ∼10^8^ to 10^5^ cfu/ml when paired with *Flectobacillus* (Supplementary Figure 5B). Despite the loss or reduction of *Acinetobacter* after kanamycin addition, overall colony-forming units in both treatments changed little because of the high abundance of *Sphingobacterium* or *Flectobacillus* which remained at densities of 10^8^–10^9^ cfu/ml (Supplementary Figure 5B). However, the loss or reduction of *Acinetobacter* significantly affected *A. aegypti* larvae, which exhibited longer development times and smaller adult sizes (Figures 3C,D). For example, even partially reducing the *Acinetobacter* titer at time 0, day 2, or day 4 when paired with *Flectobacillus* still increased development time by 26, 15, and 5%, respectively, relative to untreated *Acinetobacter*–*Flectobacillus* cultures (5.22 ± 0.1 days). The use of the antibiotic kanamycin was the only method available to selectively kill *Acinetobacter*; however, we cannot fully eliminate the possibility that kanamycin directly inhibits mosquito development rather than the elimination of *Acinetobacter*. That said, larvae treated with kanamycin still developed into adults. Further, *Escherichia* and *Rahnella* may act to slow larval development; however, this experiment did not directly test this hypothesis and future studies should be performed to identify if certain microbes have growth-inhibiting effects on *A. aegypti*.

### Dead Bacterial Amendments Also Affect Mosquito Fitness

**FIGURE 4:**
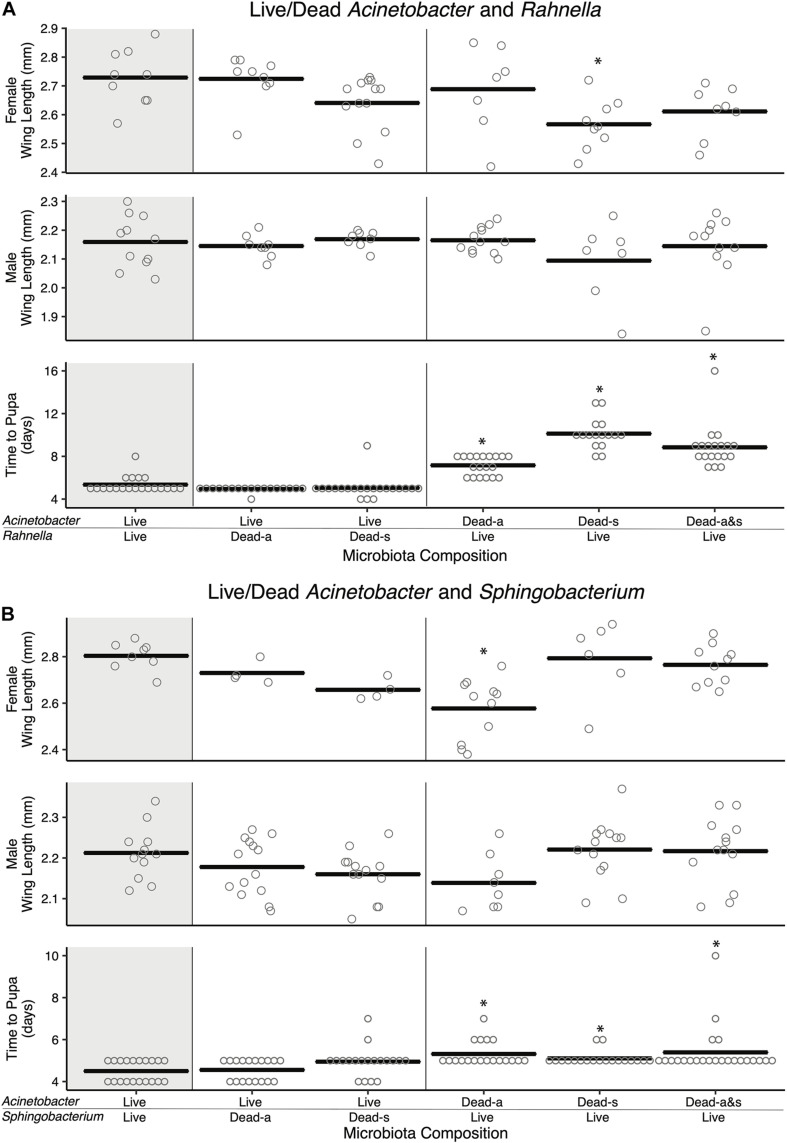
Amendment with dead bacterial cell components shows species-specific and context-dependent ability to restore adult size and development time. Pairwise combinations of living and dead cells were performed for two sets of bacteria: **(A)** *Acinetobacter* and *Rahnella*, and **(B)** *Acinetobacter* and *Sphingobacterium*. Bars indicate mean abundance for the treatment and asterisks indicate a significant difference from control (paired living bacterial microbiota) by Dunnett’s test for wing length and Steel’s test for pupation time. Dead-a (autoclaved) or Dead-s (sonicated and 0.2 μm filtered) cells were added to living cultures (Live).

Previously, axenic larvae were shown to not grow beyond the first instar when dead microbes were added to RCM and other nutrient-rich laboratory diets under standard rearing conditions (27°C and photoperiod (16 h light:8 h dark) (Valzania et al., 2018a). However, adding dead microbes to cultures did cause first instars to live longer than unfed larvae or larvae fed RCM diet alone, which suggested dead microbes provide nutrients that extend the longevity of larvae (Valzania et al., 2018b). We thus revisited our previous two-member community experiments where larvae were fed FF diet, and asked if adding one species that was dead enhanced development of larvae into adults if the second species is viable. Adding living *Acinetobacter* to *Rahnella* or *Sphingobacterium* that were killed by autoclaving or sonication resulted in larval development times that did not differ from cultures inoculated with living *Acinetobacter* and *Rahnella* or *Sphingobacterium* (Figure 4). However, adult sizes trended smaller in cultures containing one living and one dead bacterium (e.g., female A[L]-Sp[s] 2.73 ± 0.02 mm, A[L]-Sp[a] 2.66 ± 0.02 mm, A[L]-R[a] 2.72 ± 0.03 mm, A[L]-R[s] 2.64 ± 0.03 mm) vs. cultures where both bacteria were living (A[L]-Sp[L] 2.8 ± 0.02 mm; A[L]-R[L] 2.73 ± 0.03 mm) (Figure 4). In reciprocal experiments, time to pupation was significantly longer when larvae were inoculated with living *Rahnella* or *Sphingobacterium* regardless of whether dead *Acinetobacter* was heat-inactivated or sonicated (Figure 4).

Addition of autoclaved *Acinetobacter* to living *Sphingobacterium* resulted in longer development times, significantly smaller females, and smaller males, whereas sonicated *Acinetobacter* restored development times and adult sizes to levels that were similar to larvae inoculated with living *Sphingobacterium* and *Acinetobacter* (Figure 4). Adding living *Rahnella* plus autoclaved *Acinetobacter* produced larger females, whereas adding sonicated *Acinetobacter* produced smaller females than cultures inoculated with living *Rahnella* and *Acinetobacter* (Figure 4). We also assessed whether increased resources associated with adding a dead bacterium affected the population of the second living bacterium. Results showed that *Acinetobacter* populations increased less when dead *Rahnella* or *Sphingobacterium* were added than when these species were added as living bacteria (Supplementary Figure 6). There was also little change in *Rahnella* and *Sphingobacterium* abundances between living–living or living–dead combinations (Supplementary Figure 6). We thus concluded that some combinations of living and dead bacteria mimicked outcomes when both community members were viable, but most combinations did not.

### ALL7 and Endemic Microbiotas Generally Failed to Support *A. aegypti* Development When Larvae Were Fed Plant-Based Natural Diets

As previously noted, while RCM, FF, and other diets used to rear mosquitoes in the laboratory are nutrient rich, larvae feed upon detritus in the field, which primarily consists of plant debris that is comparatively nutrient poor (Anderson et al., 2016). However, many bacteria grow in environments that have nutrients that are inaccessible to animals because of their diverse catabolic metabolisms and biosynthetic abilities to produce essential amino acids, vitamins, and other factors required for growth. We thus assessed how six gamma-irradiated plant-based diets (Supplementary Table 3) affected microbiota growth and the development of *A. aegypti* larvae into adults. Results showed that the ALL7 microbiota grew to a similar density (∼10^8^ cfu/ml) in the six plant-based diets (with or without mosquito larvae) as previously observed for the RCM or FF diets (compare Supplementary Figure 7 with Figure 4). Regardless of the plant-based diet provided, most *A. aegypti* larvae did not develop into adults when either the ALL7 microbiota was added or when the endemic microbiota was added to the Tree Hole or Discarded Tire diets (Supplementary Table 3). Tulip tree (*Liriodendron tulipifera*) leaves with the ALL7 microbiota supported development of 4/20 larvae into adults, but development times were much longer (>18 days to pupa, $\bar{x}$ = 21.5 days) relative to larvae fed RCM or FF diet. The one adult female and three males that eclosed were also much smaller (Supplementary Table 4). The endemic microbiotas from the Discarded Tire and Tree Hole resulted in slightly more larvae developing into adults (3/40 larvae) than the ALL7 microbiota (0/40) but also exhibited long development times and small adult sizes (Supplementary Tables 3, 4). Thus, the six plant-based diets largely failed to support development of *A. aegypti* larvae despite growth of the ALL7 microbiota to a similar density as observed when fed laboratory diets.

### Adding RCM Components to Detritus Promotes *A. aegypti* Development

Several mosquito species have been observed to exhibit higher growth rate, survivorship, and adult size when plant detritus, as commonly fed upon by mosquito larvae in the field, is supplemented with animal tissues that contain higher amounts of protein (Yee and Juliano, 2006; Yee et al., 2007). We therefore added two RCM components: (1) lactalbumin that provides protein or (2) torula yeast that provides protein plus other macro- and micronutrients. These components were provisioned at two concentrations (1×, 2×) to the Discarded Tire detritus. We then assessed effects on microbial and *A. aegypti* growth in cultures containing the ALL7 microbiota, *Acinetobacter* alone, or *Rahnella* alone. Supplemented alone, lactalbumin, torula yeast, and Discarded Tire detritus served as low-nutrient dietary controls, while RCM diet served as a high-nutrient dietary control.

**FIGURE 5:**
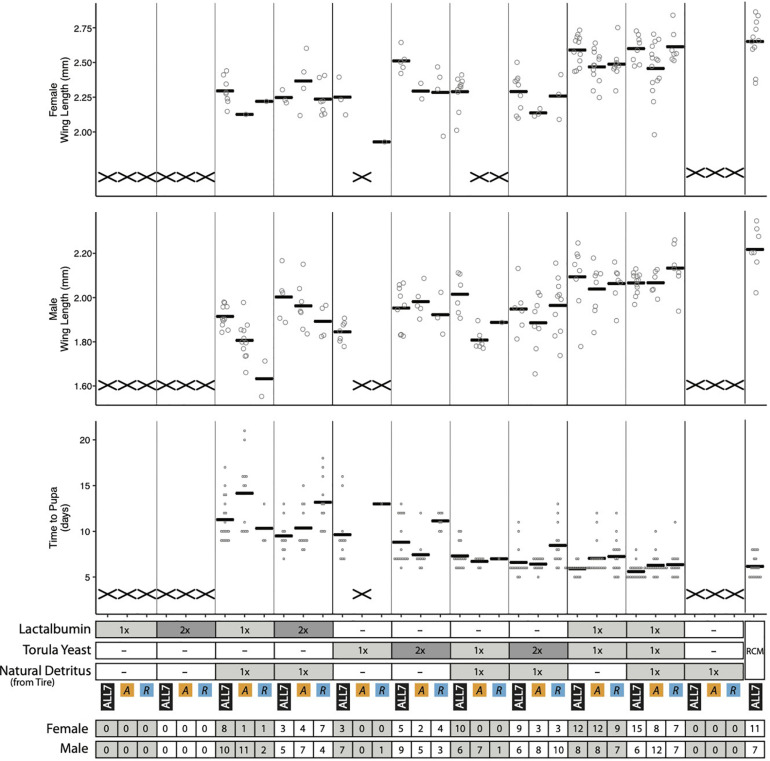
Performance metrics of *A. aegypti* reared on diet combinations of lactalbumin, torula yeast, and natural detritus. For each diet composition, mosquitoes were reared with three microbiotas: ALL7, *Acinetobacter* (A), and *Rahnella* (R). Bars indicate mean abundance for the treatment. The “X” marks at the bottom of data columns for “Time to Pupa” and “Wing Length” indicate that larvae did not develop into pupae or adults, respectively. Positive control mosquitoes were reared on RCM diet with the ALL7 microbiota. 1×, diet provisioned at 1/3 quantity of RCM diet; 2×, diet provisioned at 2/3 quantity of RCM diet; –, omitted from diet. The number of adult male and female mosquitoes is listed at the bottom for each treatment.

Assessment of bacterial growth indicated that the ALL7 microbiota and *Rahnella* alone grew to ∼10^8^ cfu/ml across most treatments with the exception of cultures containing only lactalbumin where densities were >10^7^ cfu/ml (Supplementary Figure 8). Monoxenic *Acinetobacter* treatments in contrast grew to < 10^7^ cfu/ml in all treatments except with RCM diet (Supplementary Figures 5, 8). Assessment of mosquito growth showed that no larvae developed into adults when fed lactalbumin or tire detritus alone, but adding lactalbumin to tire detritus plus the ALL7 microbiota resulted in most larvae developing into adults (Figure 5). Lower proportions of larvae developed into adults when lactalbumin was added to tire detritus in monoxenic *Rahnella* or *Acinetobacter* treatments (Figure 5). Surviving progeny across these treatments exhibited longer development times and smaller adult sizes when compared with progeny from the positive control (Figure 5).

Half of the progeny fed 1× torula yeast alone developed into adults in the presence of the ALL7 microbiota (10/20), whereas almost no progeny developed into adults when fed 1× torula yeast in cultures containing monoxenic *Rahnella* (1/20) or *Acinetobacter* (0/20) (Figure 5). Increasing the amount of torula yeast to 2× resulted in most progeny developing into adults when the ALL7 community was present (14/20), whereas only ∼40% of larvae developed into adults in cultures containing monoxenic *Rahnella* (7/20) or *Acinetobacter* (7/20). Similar to RCM-fed larvae, most progeny developed into adults regardless of the microbiota composition (ALL7, *Rahnella*, *Acinetobacter*) when fed a mix of torula yeast and lactalbumin (with or without the addition of detritus) (Figures 1, 5). Development times and adult sizes for treatments fed a mix of lactalbumin and torula yeast were also similar to progeny from the RCM diet control (Figures 1, 5). Taken together, addition of a single protein (lactalbumin) to plant-based detritus strongly promoted development of *A. aegypti* into adults in the presence of the ALL7 microbiota, whereas addition of mix of proteins and micronutrients (torula yeast) to either detritus or lactalbumin promoted development in the presence of ALL7, *Rahnella*, or *Acinetobacter*.

## Discussion

The goal of this study was to characterize general patterns in regard to whether (1) microbes that promote growth of *A. aegypti* when fed nutrient-rich RCM diet similarly promote growth when larvae are fed other diets, and (2) single species of bacteria similarly or differentially support larval growth when compared with conventional or simplified communities of bacteria. Overall, our results suggest that the growth-promoting effects of microbes vary with nutrient environment. For larvae fed nutrient-rich RCM and FF diets, our results corroborate previous findings showing that axenic larvae require living bacteria for growth, while no differences in development time or adult size were detected between larvae that were reared with an ALL7 vs. a conventional microbiota. However, experiments using individual or paired members of the ALL7 community revealed differences between RCM and FF diet in the growth-promoting potential of particular bacteria (Figure 1). For larvae fed plant-based detritus, axenic larvae also do not grow. ALL7 and endemic microbiotas grow to comparable densities in water containing detritus diets as observed with laboratory diets (Supplementary Figures 1A,2,7,8), but largely do not support larval growth into adults unless additional protein or yeast is added (Figure 5).

Microbiota composition in *A. aegypti*, like other mosquito species, varies greatly between populations (Coon et al., 2016b; Muturi et al., 2016b; Dickson et al., 2017; Thongsripong et al., 2018). In turn, no core microbiota consisting of particular genera or species has been identified in *A. aegypti* or other mosquito species although bacteria in certain higher order taxa such as Actinobacteria, Sphingobacteria, and Gammaproteobacteria are commonly present (Coon et al., 2016b; Dickson et al., 2017; Thongsripong et al., 2018). Variability in microbiota composition further mirrors data showing that the microbial communities in the aquatic habitats where mosquitoes develop are also highly variable in composition, and that most microbes detected in larvae from a particular collection site and date are also present in the aquatic habitat from which they came (Strand, 2018).

The species of bacteria we selected for inclusion in the ALL7 community are known gut community members in *A. aegypti* but could also be distinguished from one another by colony morphology and growth dynamics which made it easier to monitor their abundance in the water where larvae feed. Larvae failed to grow when fed RCM diet axenically but had very similar developmental rates and adult sizes when fed RCM diet plus a conventional community, the ALL7 community, or most individual members of the ALL7 community (Figure 1). These results corroborate previous findings that larvae require microbes for development under standard rearing conditions but that this requirement is not species specific (Coon et al., 2014, 2016b). In contrast, while having similar overall abundances in rearing water (Supplementary Figures 1, 2), no single bacterial species supported larval growth rates or adult sizes equal to that of the ALL7 community in mosquitoes fed FF diet (Figure 1), suggesting an important role for microbe–microbe interactions in this nutrient environment. These results also suggest nutritional differences exist between RCM and FF diets despite their overlap in macronutrient ingredients (Supplementary Table 2). The observation that conventional and ALL7 communities grow to densities between 10^7^ and 10^9^ cfu/ml in cultures fed plant-based detritus or laboratory diets (i.e., RCM, FF) indicates that nutrients are sufficient to comparably support microbial growth (Supplementary Figures 1A,7). On the other hand, the failure of most larvae to develop into adults indicates that plant-based detritus and the bacteria present in these cultures provide inadequate resources for *A. aegypti* (Figure 5). One of these inadequacies is insufficient protein since adding lactalbumin to cultures containing tire detritus plus the ALL7 community substantially rescues larval development into adults (Figure 5). This outcome also supports the previous suggestion that animal detritus can be an important source of nutrition for mosquito larvae in the field (Yee and Juliano, 2006; Yee et al., 2007). However, it is also possible other species of bacteria or other microorganisms provide essential resources when only plant-based detritus is available. One candidate that could be especially important are yeasts and other fungi, which have been identified in the aquatic habitats and microbiotas of several mosquito species (Chandler et al., 2015; Muturi et al., 2016a; Steyn et al., 2016). Results of this study also support this suggestion since adding heat-killed torula yeast to cultures containing the ALL7 community promotes larval growth into adults.

While a few studies identify negative effects of microbiota diversity on multicellular animals (Krams et al., 2017; Napflin and Schmid-Hempel, 2018), most studies identify benefits with increased diversity for both invertebrates (Newell and Douglas, 2014; Callens et al., 2018; Gould et al., 2018) and vertebrates (Knutie et al., 2017; Ellison et al., 2019). Increased diversity can create novel microbe–microbe interactions that change gene expression in the overall microbial community or in particular community members, which can result in emergent properties that change the overall metabolism or ecological interactions among microbial species (Ibberson et al., 2017; Kešnerová et al., 2017; Gould et al., 2018). Previous studies with mosquitoes identify conditions where more diverse microbial assemblages promote survivorship and development (Díaz-Nieto et al., 2016; Travanty et al., 2019). In contrast, our results suggest the benefits of increasing microbiota diversity for larval growth rates and adult size in *A. aegypti* are contextual, with diet being a key variable as to whether increased diversity promotes development or not. While we observed striking variation in growth-promoting activity of different microbes in the FF diet assays, all species in our ALL7 community grew to similar densities across all diets we tested (Supplementary Figure 2) and there was no clear relationship between higher density and increased mosquito development. For example, *Microbacterium* grew to densities near 10^9^ cfu/ml but was unable to support mosquito development, whereas *Acinetobacter* grew to a density near 10^7^ cfu/ml in RCM diet which enabled full mosquito development (Supplementary Figure 2). This further suggests that currently unknown traits in particular species of bacteria (e.g., vitamin production) found in association with *A. aegypti*, rather than differences in abundance, are important for larval growth. Microbial growth rate and turnover or the digestibility of a microbe might be possible features that affect mosquito nutrient acquisition and development. Our finding that certain two-member microbial communities promote growth rates and adult sizes that are very similar to the ALL7 community when larvae are fed FF diet (Figure 2) further indicate that even extremely simple communities can generate emergent properties under certain dietary conditions that enhance larval performance.

Developmental differences among insects and other multicellular animals can result from absent or imbalanced nutrients including inorganic micronutrients (e.g., salts, trace metals), amino acids found in protein, and sterols which most bacteria cannot synthesize (Huang and London, 2016; Wei et al., 2016) but may be accessible from plant and animal tissues, or microbial eukaryotes including fungi (Baker, 1992; Gray et al., 2006; Piper et al., 2014; Simpson et al., 2017). Our studies using FF diet indicate that two-member communities containing *Acinetobacter* promote larval growth comparably to the ALL7 community (Figure 2 and Supplementary Figure 9), while providing one heat-killed member in a two-species community also comparably supports larval growth in some cases (Figure 4). This result is consistent with the suggestion that microbes, in part, serve as food for *A. aegypti* but the inability of dead bacteria to support development under a typical light:dark photoperiod indicates that larval growth also involves factors that living microbes provide. Similar to the results from Keebaugh et al. (2018), our assays using detritus diets also indicate that adding dead torula yeast to cultures containing living bacteria likely provides macronutrients like protein plus micronutrients such as vitamins, sterols (e.g., ergosterol), and trace metals that enable larvae to develop without any detritus (Figure 5). In contrast, lactalbumin and detritus are unable to support larval growth into adults when supplied individually with living bacteria but do support growth to the adult stage when provisioned together. This complementarity potentially stems from the juxtaposition of trace metals, salts, and plant sterols (e.g., sitosterol, stigmasterol), which are common in natural detritus, with added protein from lactalbumin plus other unknown factors provided by viable ALL7 community members. The recent report that *A. aegypti* can be reared axenically only when fed a high-nutrient diet with very high densities of autoclaved bacteria (Correa et al., 2018) further suggests that living microbes may provision factors, in addition to macronutrients, that are at extremely low concentrations in dead bacteria or that rapidly degrade after bacterial death.

In natural mosquito habitats, microbe populations experience events that alter community diversity and abundance (e.g., drought, algal blooms, agricultural runoff). Microbes also rapidly transit the larval gut in association with feeding (Allison and Martiny, 2008; Green et al., 2008; Coon et al., 2017). Thus, mosquito larvae may experience frequent changes in microbiota composition as a function of environmental conditions or foraging activity in the water column that can disrupt development. Alternatively, this rapid turnover of the microbiota may enable larvae to select and consume advantageous microbe communities. Stochastic variation in the microbial community of our insectary’s rearing water over 6 months was enough to change host development (Supplementary Figure 1C), underscoring the potential for natural microbial variation to affect wild mosquito populations. More generally, changes in the environmental or larval gut microbial communities may have cascade effects on mosquitoes that extend to their adult life phase and have consequences for disease transmission.

Using *Drosophila melanogaster* as a model for host–microbe interactions, researchers have identified numerous growth-promoting factors produced by the microbiota, which include structural components of the cell membrane and catabolic and anabolic metabolites (acetic acid, ribonucleotides, vitamin co-factors) (Blatch et al., 2010; Shin et al., 2011; Storelli et al., 2011; Piper et al., 2014; Matos et al., 2017; Sannino et al., 2018; Consuegra et al.,2020a,b). Among these factors, B vitamins are cofactors critical to the central metabolism of animals (tricarboxylic acid cycle; aerobic respiration; metabolism of amino acids, fatty acids, DNA), but cannot be synthesized by animals and must be obtained from the diet or supplied by microbes inhabiting the animal body (Douglas, 2017). Riboflavin (Wong et al., 2014), thiamine (Sannino et al., 2018), and pantothenate (Consuegra et al.,2020a,b) have been demonstrated to be supplied by the microbiota and directly influence growth in *D. melanogaster* larvae, a terrestrial species. However, microbiota-produced B vitamins may be more important to aquatic animals like mosquito larvae because of their instability in liquid media, where light, oxygen, high temperature, or changes in pH can rapidly degrade them into forms that cannot be used (Sheraz et al., 2014; Schnellbaecher et al., 2019). Further, work using holidic diets that excluded individual micronutrients showed direct evidence that microbes promote the growth of mosquito larvae through the production of various B vitamins (Wang et al., 2021). In addition, this work suggests that completely axenic rearing of mosquito larvae was previously thwarted by the degradation of B vitamins via photodegradation in the aquatic rearing environment (Correa et al., 2018; Wang et al., 2021).

A major pattern observed across the experiments performed in this study was that mosquito larvae reared with individual bacteria grew slower and to smaller adult sizes than those reared with two or more bacterial species, suggesting there is a benefit to harboring a more diverse microbiota. This pattern follows theoretical predictions (Lozupone et al., 2012; Friedman et al., 2017) and has also been observed in other systems (Newell and Douglas, 2014; Knutie et al., 2017; Callens et al., 2018; Gould et al., 2018; Ellison et al., 2019; Consuegra et al.,2020a,b); however, the underlying mechanisms driving this process are less understood. Recently, it was shown that the exchange of metabolites between *Acetobacter pomorum* and *Lactobacillus plantarum*, common members of the *D. melanogaster* microbiota, induced the production of B vitamins and cofactors that is not observed in the monoculture of either species, which resulted in increased larval growth (Consuegra et al., 2020a). This emergent property of co-culturing promoted *D. melanogaster* larval growth even in a low nutrient diet and demonstrates how cross-feeding (syntrophic) interactions, especially involved with vitamin biosynthesis, can result in major changes to host animal growth. Environmental multi-species communities (18 species) of non-host-associated bacteria have also been shown to depend on syntrophic interactions to share B vitamins and their precursor metabolites, with all members relying on a shared pool of micronutrients to survive (Romine et al., 2017). Altogether, there are data coming from both host–microbe interaction studies and microbial community ecology that suggests the biosynthetic repertoires (particularly in B vitamins) of interacting bacteria may dramatically alter the nutritional composition present in an environment, which in turn affects host nutrition and growth.

## Conclusion

Our results indicate that growth of *A. aegypti* larvae is strongly affected by both microbes and diet. While our work used only one strain of *A. aegypti* (UGAL), it seems likely that developmental responses to macronutrients may be conserved in mosquitoes and research on different species will be very informative. Moving forward, defined (holidic) diets will be required to determine how individual microbial species alter larval homeostasis and to identify specific nutrients or growth-promoting factors produced by viable microbes. These rearing techniques will further facilitate comparative studies, such as identifying differences in microbiota-based nutrient requirements among mosquito species and how these differences influence mosquito life-history ecology (e.g., container vs. running-water breeders; detritivorous vs. carnivorous larvae) and competition between mosquitoes in natural habitats. Better understanding of host–microbe interactions in mosquitoes is important because of the implications for vectoring human disease; however, mosquitoes also present a powerful system for studying the effects of microbiota alteration on host performance and fitness because microbes and diet can be easily manipulated in their aquatic rearing arrangement. Finally, assessing microbial interactions within the microbiota and subsequent changes in metabolic networks, niche partitioning, and resource allocation will be generally informative to microbial community assembly and stability in host-associated ecosystems.

## Materials and Methods

### Mosquitoes and Diets

UGAL *Aedes aegypti* were originally collected in Athens, GA (Valzania et al., 2018b). Non-sterile (conventional) larvae were reared at 27°C under a 16 h light:8 h dark photoperiod in 2-L pans containing water and fed RCM diet which contained equal parts (w/w) powdered rat chow diet (LabDiet 5012, St. Louis, MO, United States), heat-killed torula yeast (Frontier Scientific Services, Newark, DE, United States), and lactalbumin (Sigma, St. Louis, MO, United States) (Coon et al., 2014; Bond et al., 2017). Larvae can also be reared by feeding them FF diet which consisted of TetraColor Tropical Granules (Tetra, Blacksburg, VA, United States). Adults of both sexes were maintained in Plexiglas cages at 27°C and a 16 h light:8 h dark photoperiod and fed 10% sucrose (w/v) in water. Adult females laid eggs after consuming commercially purchased rabbit blood (Hemastat Laboratories, Dixon, CA, United States) using artificial feeders. For experiments, larvae were maintained under the same physical conditions as our conventional culture and fed RCM diet, FF diet, or plant-based natural diets collected from locations near the University of Georgia that consisted of leaves, leaf litter, or wet detritus from a sweet gum (*Liquidambar styraciflua*) tree hole or a discarded automobile tire (Supplementary Table 3). Any invertebrates including mosquito larvae were removed from these materials to minimize the possibility of animal tissue contamination before drying at 60°C for 48–72 h. Each material was then ground into a fine powder with a blade grinder (Hamilton Beach, Glen Allen, VA) followed by sterilization via gamma irradiation at 10 kGy as previously described (Coon et al., 2014). Endospores of *Bacillus* species have been shown to be reduced 10-fold when exposed to ∼2 kGy of gamma radiation (Cote et al., 2018). The 10 kGy used on our diets would reduce the spore population by approximately 10^5^. Our main laboratory cultures as well as all experiments were maintained at 27°C under a 16 h light:8 h dark photoperiod.

### Bacterial Isolates

Water samples were collected from two rearing pans containing fourth instar UGAL *A. aegypti* and four outdoor containers containing mosquito larvae that were located within 2 km of the laboratory in the fall of 2017 (Supplementary Table 1). Larvae and organic debris (e.g., leaf tissue) were first removed from samples to minimize carryover of potential nutrients. The microbial communities in each water sample were next centrifuged at 6,000 rpm (rad) for 15 min. The resulting pellets were then resuspended in a 1:1 mixture of sterile glycerol:1× PBS and cryopreserved at –80°C. Strains of bacteria from the laboratory or field collection sites were isolated on minimal medium of 1/10 diluted 869 agar plates (Eevers et al., 2015). Unique colony morphologies were selected and passaged three times to new agar plates to ensure individual isolates. Isolates were then suspended in a 1:1 mixture of sterile glycerol:1× PBS and cryopreserved at -80°C. Template DNA was extracted from each isolate with the DNeasy Blood and Tissue kit (Qiagen, Valencia, CA, United States) and used to amplify a portion of the 16S rRNA gene with the primer set 27fshort-1507r, HotMaster Taq DNA polymerase (Quantabio, Beverly, MA, United States), and previously described PCR conditions (Martinson et al., 2011). Amplicons were visualized on a 1% agarose gel and cleaned with the QIAquick PCR purification kit (Qiagen, Valencia, CA, United States) before submitting for Sanger sequencing at Eurofins Genomics (Louisville, KY, United States). Isolates were identified to genus by blasting the 16S rRNA sequence to the NCBI nr database.

Six isolates were selected as representatives of environmental microbes that were found in water that contained wild or laboratory-reared mosquito larvae (Supplementary Table 1). These bacterial species were selected based on (1) the ability to grow relatively quickly on a common medium (1/10 diluted 869 agar), (2) the ability to differentiate species by colony morphology (size, margin shape, color), and (3) that they were a taxonomically diverse set of species. Colony morphology for each bacterium at ∼24, ∼48, and > 48 h growth (on 1/10 diluted 869 agar) was used to differentiate species when they were grown in two-member communities and images of these differences can be found in Presentation S1. Bacterial isolates were assayed for resistance to antibiotics to help design the experiments presented in Figures 3B,D using 1/10 diluted 869 agar plates with kanamycin (50 μg/ml), ampicillin (100 μg/ml), spectinomycin (50 μg/ml), chloramphenicol (25 μg/ml), and tetracycline (10 μg/ml) (Supplementary Table 1). We also selected *Escherichia coli* K-12 substr. MG1655 because this species is a known gut community member in *A. aegypti* (Thongsripong et al., 2018) and was also used previously in gnotobiotic rearing assays (Coon et al., 2014, 2017; Valzania et al.,2018a,b). The combination of these seven bacterial species was designated the ALL7 microbiota. To obtain dead cell additives, bacteria were grown in 1/10 diluted 869 liquid media to near stationary phase. A dilution series was performed to determine the colony-forming units per milliliter for subsequent concentration to 10^9^ cfu/ml. Bacteria were pelleted at 2,000 × *g* and resuspended in sterile water to a concentration of 10^9^ cfu/ml. Dead bacteria were created in two ways: (1) autoclaved, which does not preserve certain heat-instable nutrients, or (2) sonicated and filter sterilized, which is capable of preserving heat-instable nutrients (Valzania et al., 2018b).

### Mosquito Rearing Conditions

Axenic first instars were produced by surface sterilizing eggs using previously established methods (Coon et al., 2014). Larvae were reared in two types of containers: 25-cm^2^ cell culture flasks (Corning, Corning, NY, United States) or 6-well plates that served as rearing containers (Genesee Scientific, San Diego, CA, United States). Flasks contained 20 ml of sterile water, 60–65 mg of FF diet (3–3.25 mg/larva), 20 axenic larvae, and 10 μl of a given bacterial suspension. Individual wells in culture plates contained 5 ml of water, 10 axenic larvae, 5 μl of bacterial suspension, and RCM diet on a feeding schedule (3.3 mg at hour 0, 24; 8.3 mg at hour 72, 96; total = 2.32 mg/larva) (Valzania et al., 2018b). Rearing with natural diets was also performed in 6-well plates with 5 ml water and 10 axenic larvae, but diet was added *ad libitum* to encourage larval development.

For experimental treatments, bacterial isolates were grown on 1/10 dil. 869 agar plates for 24–48 h at 37°C until individual colonies were visible to ensure single species growth. Colonies were collected off plates with sterile disposable loops (Genesee Scientific) and diluted into 1 ml of sterile 1× PBS. A dilution series was performed for each bacterium using the SP-SDS method to calculate the colony-forming units per milliliter and initial concentration of cells in each experimental treatment (Thomas et al., 2015). This serial dilution method was also used for subsequent counts of bacterial abundance.

Assays where particular bacteria were added or eliminated were conducted in 25-cm^2^ flasks using the aforementioned methods and microbiota composition/abundance was monitored by dilution series. *Acinetobacter* was selected as the target bacterium because bacterial pairs including it produced mosquitoes robust in performance metrics and it was sensitive to at least one antibiotic, unlike *Sphingobacterium* and *Flectobacillus*. Addition or removal of the second bacterium (*Acinetobacter*) occurred on days 2 and 4 of larval development. Removal of *Acinetobacter* was performed with the addition of kanamycin at 50 mg/ml, which was able to kill or severely decrease the growth of *Acinetobacter* while not inhibiting the growth of *Sphingobacterium* or *Flectobacillus*. Tests of the effects of dead bacteria on mosquito development used the 25-cm^2^ cell culture flasks with 20 axenic larvae and FF diet experimental design described previously. Living cells were inoculated at time 0 at concentrations near 10^6^ cfu/ml. Dead cell homogenates or filtrates were provisioned daily at concentrations similar to those found in living cells (following are the final concentrations in the rearing container): time 0—10^6^ cfu/ml, day 1—5 × 10^6^ cfu/ml, day 2—10^7^ cfu/ml, day 3—10^7^ cfu/ml, and day 4—10^7^ cfu/ml.

Assays where two components of the RCM diet (i.e., lactalbumin, torula yeast) were added to tire detritus were performed in 6-well plates with 5 ml sterile water, 10 axenic larvae, and 10^6^ cfu/ml of the ALL7 microbiota, *Acinetobacter*, or *Rahnella*. Detritus was added to each well on a feeding schedule (3.3 mg at hour 0, 24; 8.3 mg at hour 72, 96; total = 2.32 mg/larva). Lactalbumin and torula yeast were added to DI water at a concentration of 10 mg/ml and autoclaved before use. Each component was added to a well at 1 × (1.1 mg at hour 0, 24; 2.76 mg at hour 72, 96; total = 0.77 mg/larva) or 2 × (2.2 mg at hour 0, 24; 5.52 mg at hour 72, 96; total = 1.54 mg/larva). Provisions of the lactalbumin and torula yeast at 1 × level were equivalent to one-third the total weight of complete RCM diet (see above).

### Microbiota Composition

Each of the seven bacteria used in pairwise microbiota treatments could be distinguished from each other by colony morphology, including colony size, margin shape, and color (at 24, 48, > 48 h) (Supplementary Table 1). *Acinetobacter*, *Rahnella*, and *Microbacterium* were also readily identifiable in the ALL7 mixture due to their morphology and abundance, but the other species could not be distinguished because they were present at lower abundance. Rearing water was sampled at two timepoints during development of mosquito larvae (3 days post-inoculation; at fourth instar) to determine the microbiota composition and abundance. A dilution series was performed as described previously, and colony morphologies were observed under a dissection microscope at × 1–4 magnification. These assessments further confirmed or refuted contamination and the expected microbiota composition in each treatment. Contaminated samples were removed.

### Mosquito Fitness Measures

The time to pupation for conventionally reared *A. aegypti* fed RCM diet is 5 days. Larval development was observed daily, and the number of pupae and adults was recorded. Adults were aseptically collected, sex was determined, and wings were removed and placed onto microscope slides. Photographs of the wings were taken with a dissection microscope and wing length was measured using the LASX software (Leica Microsystems, Wetzlar, Germany). The distance between the alular notch to the apex of the radius vein 3 was measured as a well-established proxy for adult size (Yeap et al., 2013). Individual mosquitoes exposed to a given treatment served as the unit of replication when comparing development times with pupation and adult sizes between treatments. Within select treatments, development times and adult sizes were also compared between larvae that developed in different culture flasks. Statistical tests were performed in JMP pro14 (SAS Institute, Cary, NC, United States). Wing length data were analyzed by *t*-test, or ANOVA followed by Dunnett’s or Tukey’s HSD *post-hoc* comparison test. Because time to pupation was binned into 1-day intervals (non-parametric distribution), data were evaluated with Wilcoxon, Steel, Kruskal–Wallis, or Steel–Dwass tests.

## Data Availability Statement

The original contributions presented in the study are publicly available. This data can be found here: Accession numbers for the 16S rRNA gene sequences of the ALL7 microbiota members are deposited in GenBank (MN544614–MN544619) and individually listed in Supplementary Table 1. All other data are available upon request to the authors.

## Author Contributions

VM and MS designed the experiments, collected the data, and contributed to the writing of the manuscript. VM analyzed the data. All authors read and approved the final article.

## Conflict of Interest

The authors declare that the research was conducted in the absence of any commercial or financial relationships that could be construed as a potential conflict of interest.

## Abbreviations

ALL7: a simplified 7-member community of bacteria including *Acinetobacter* sp. *Sphingobacterium* sp. *Flectobacillus* sp. *Serratia* sp. *Rahnella* sp. *Microbacterium* sp. and *Escherichia coli*
**RCM**: rat chow mix (laboratory mosquito diet consisting of equal parts (w/w) rat chow heat-killed torula yeast (*Cyberlindnera jadinii*) and lactalbumin)
FF: fish food (TetraColor Tropical Granules).
