# Supplementary material for: Diet–Microbiota Interactions Alter Mosquito Development
Source: Front Microbiol. 2021 Jun 8;12:650743. doi: 10.3389/fmicb.2021.650743 (PMC8217444; doi:10.3389/fmicb.2021.650743)

Notes about this document:

- Top view = viewed from top of agar plate
- Bottom view = view from bottom of agar plate
- 0.8x or 2.0x = magnification

E. coli, K-12 subsets. MG1655

Top View

Day 1		Day 2
0.8x	2.0x	0.8x

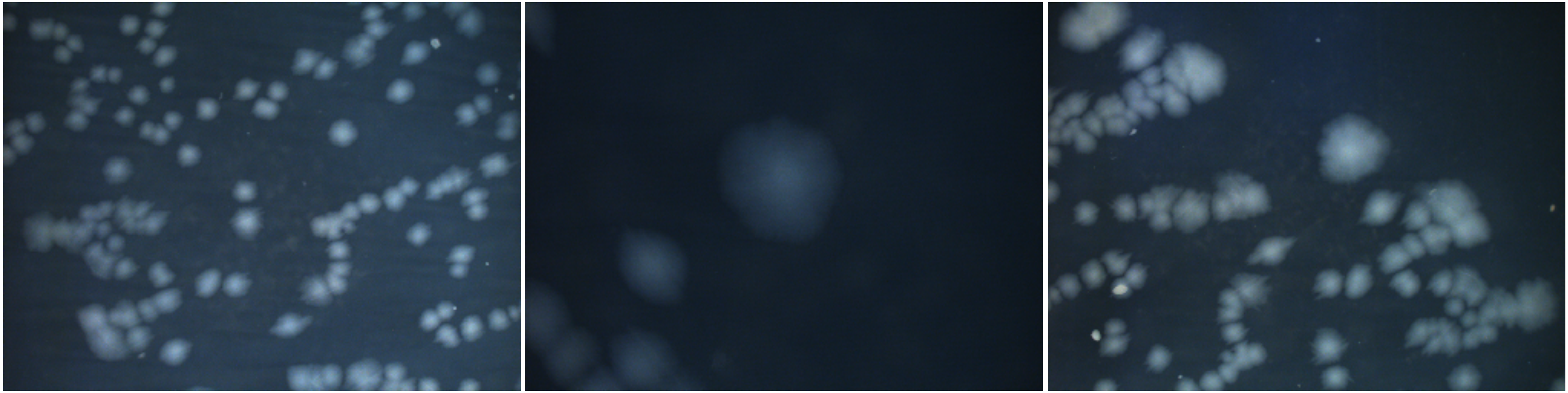

Bottom View

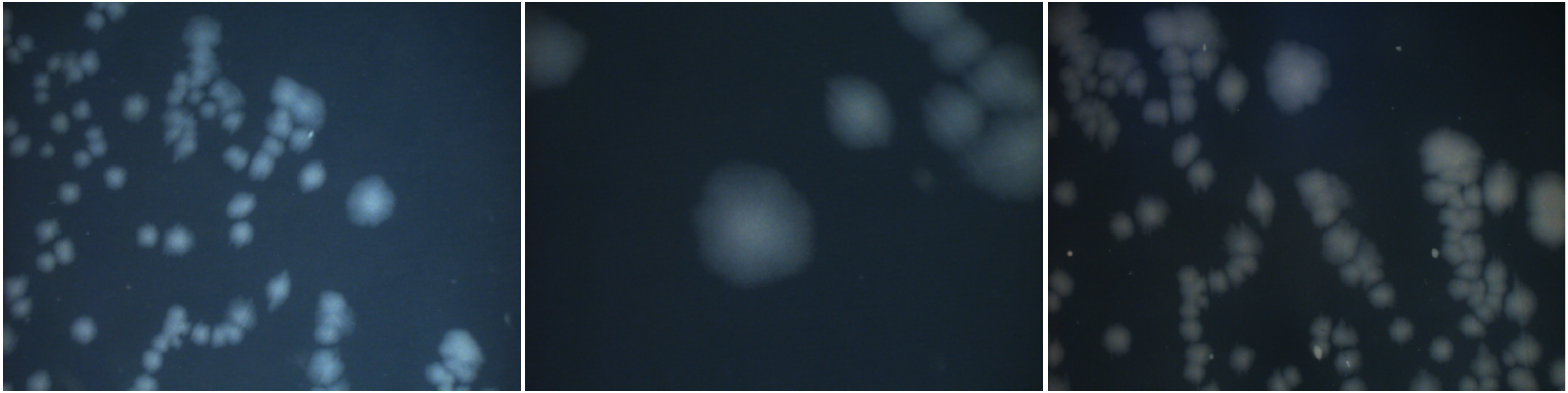

Microbacterium, Lab0001

Top View

Day 1	Day 2	Older
	0.8x	
**Pinpoint**	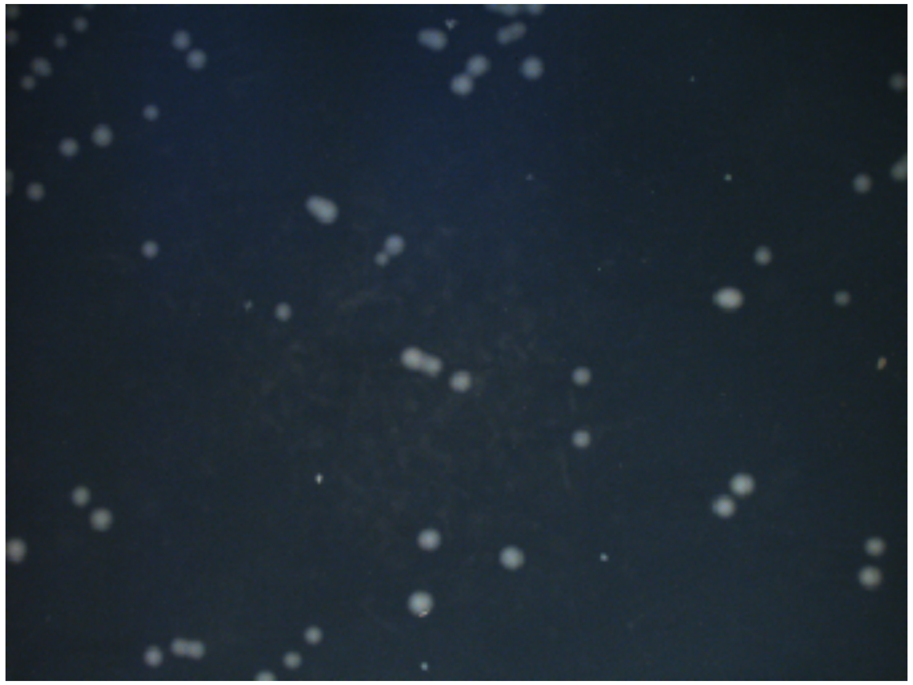	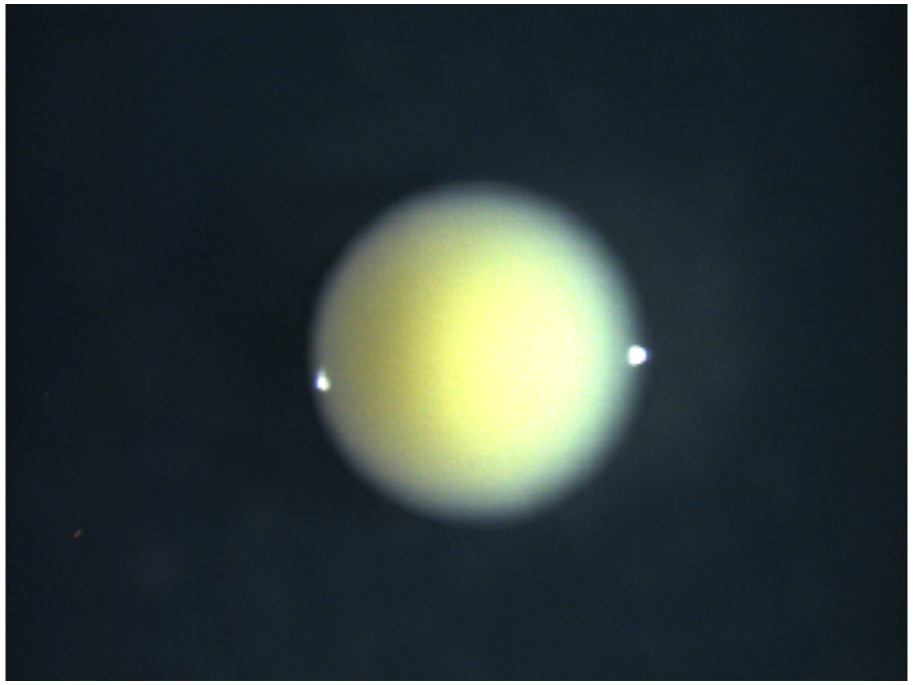

Bottom View

**Pinpoint**	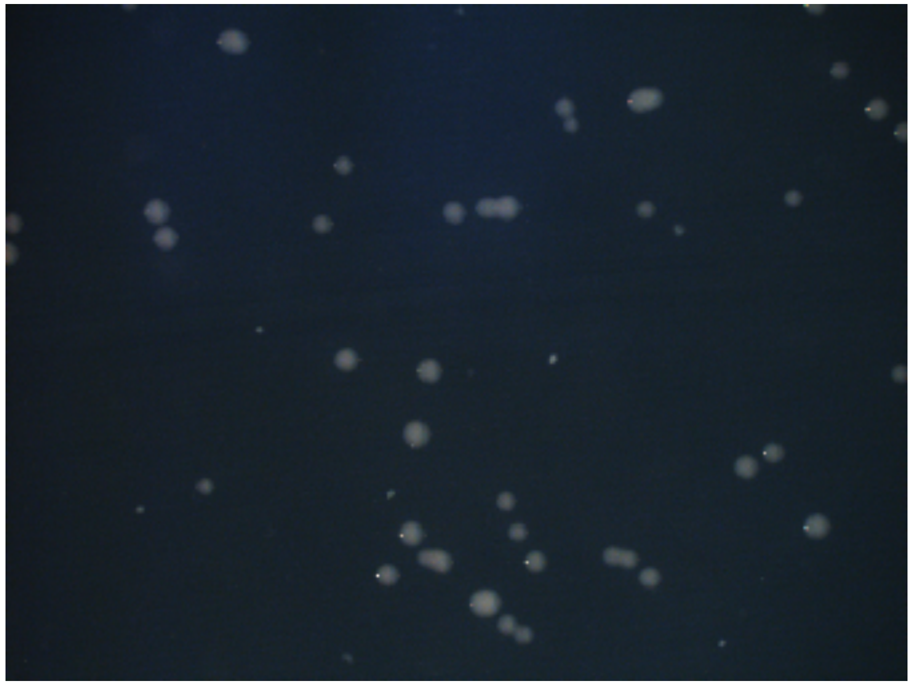	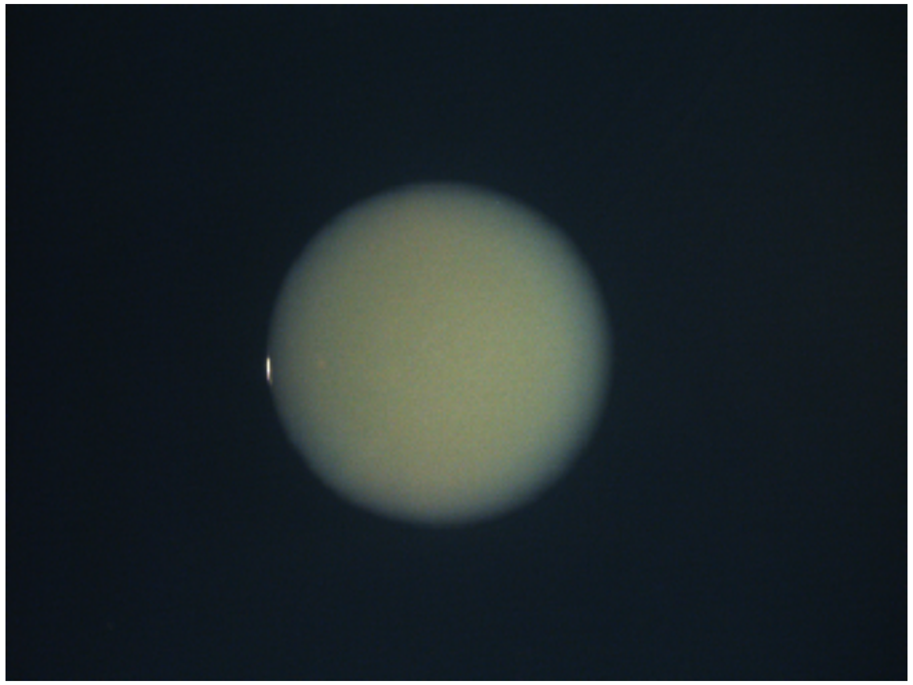

Rahnella, Rain0009

Top View

Day 1 | **Day 2**

0.8x | **2.0x** | **0.8x**

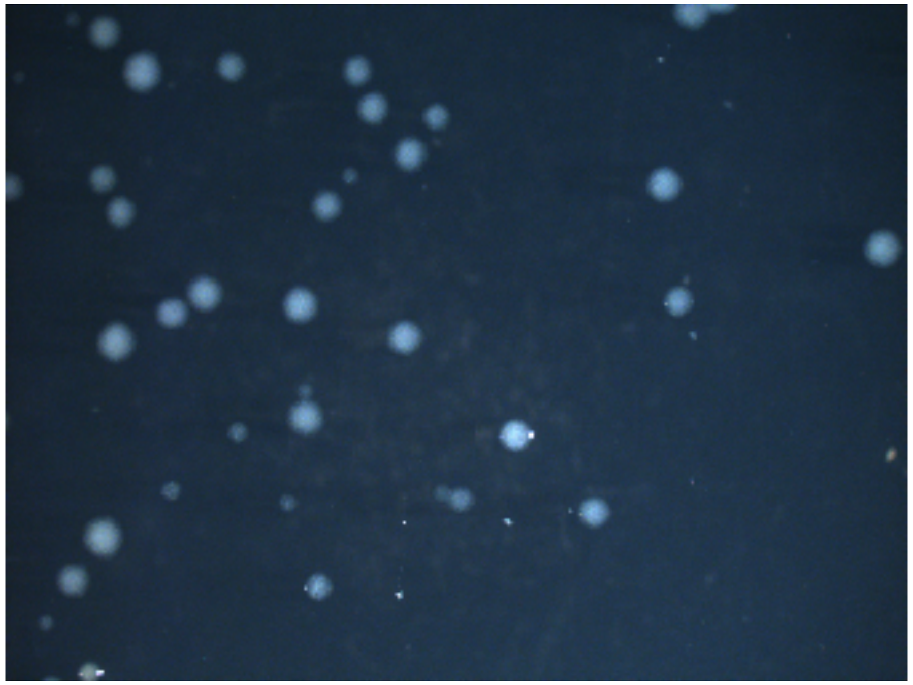

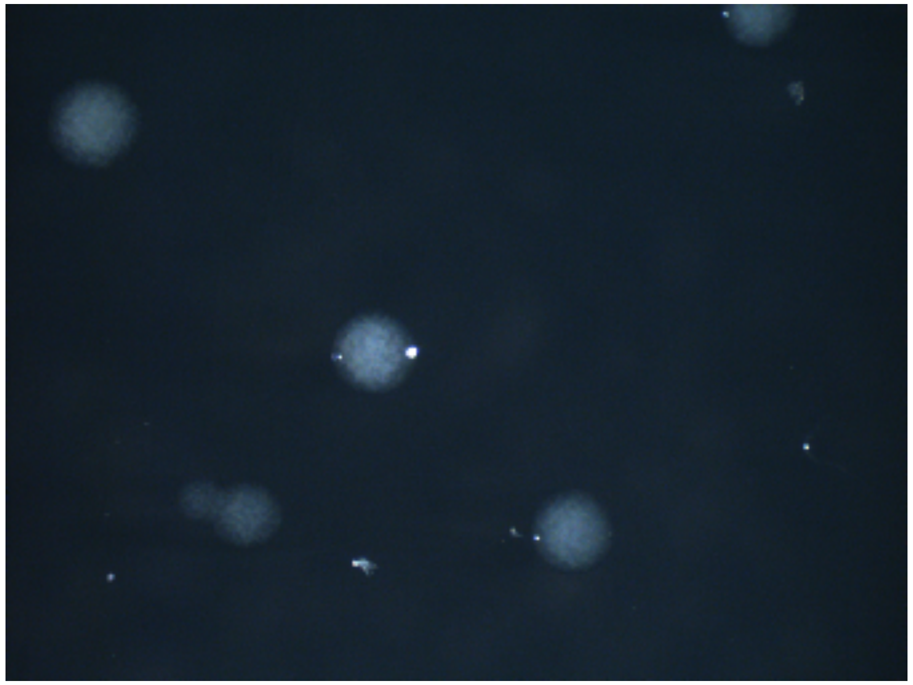

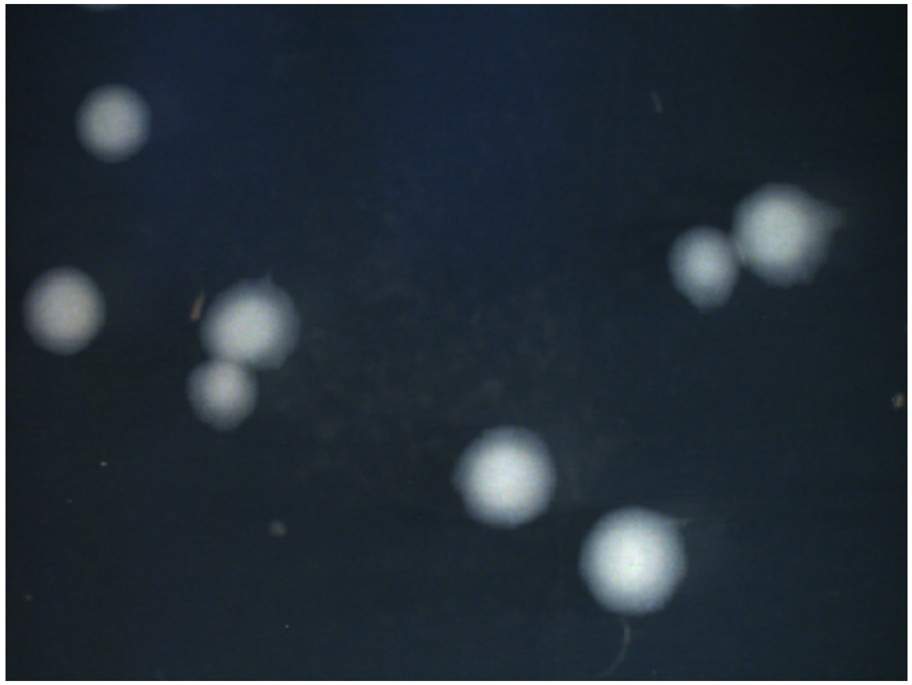

Bottom View

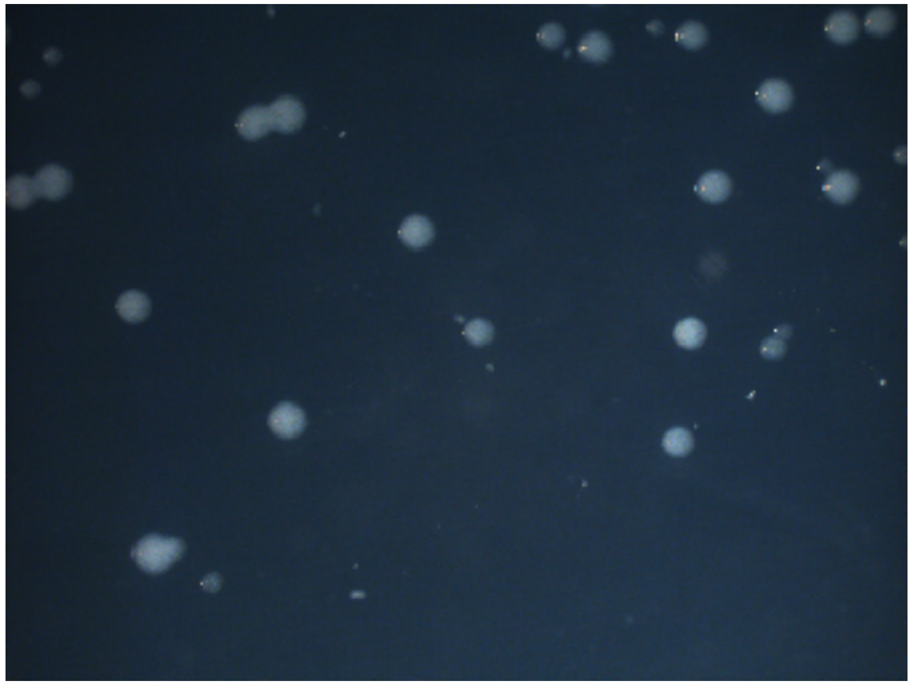

No photo

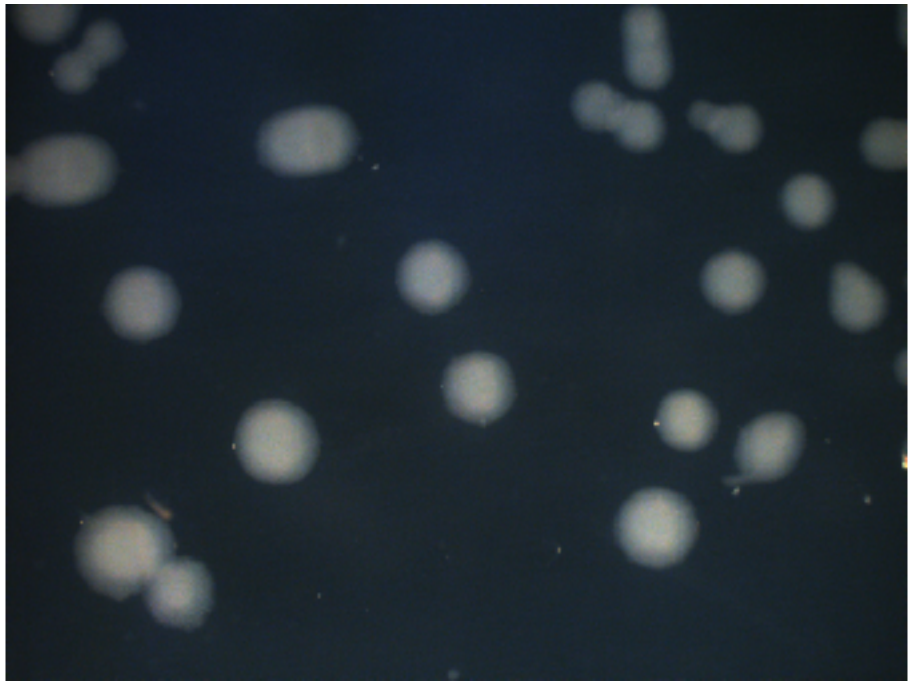

Serratia, Rain0010

Top View

Day 1	Day 2	
0.8x	0.8x	2.0x

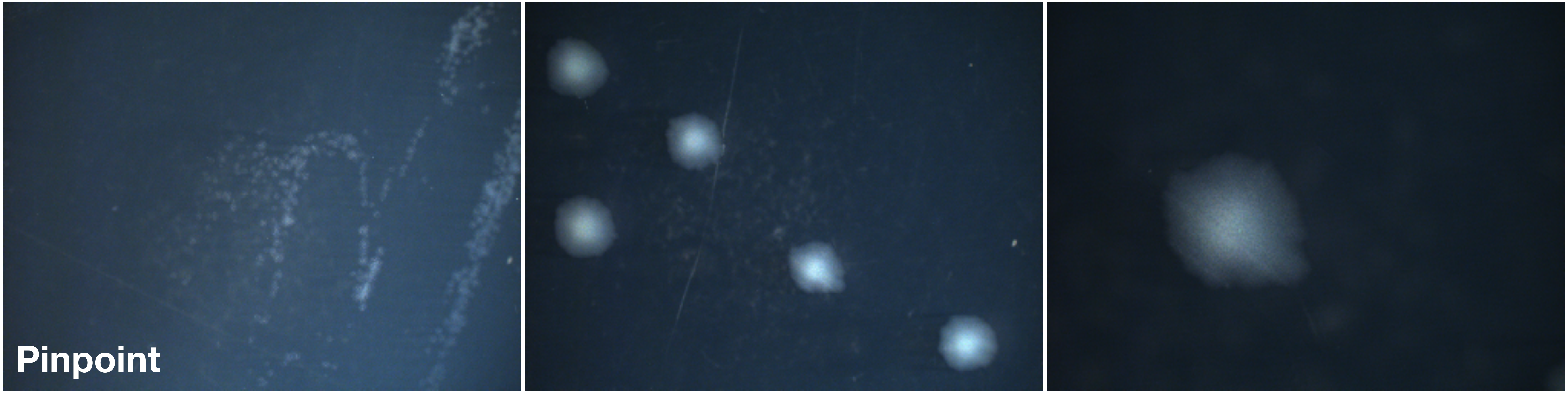

Bottom View

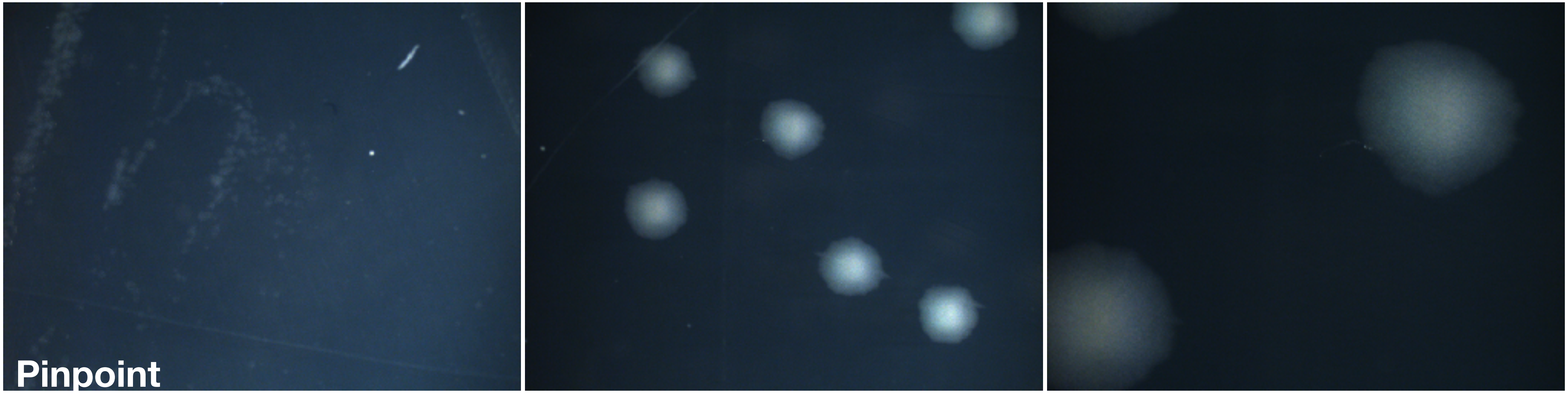

Flectobacillus, Rain0001

Top View

Day 1		Day 2
0.8x	2.0x	0.8x

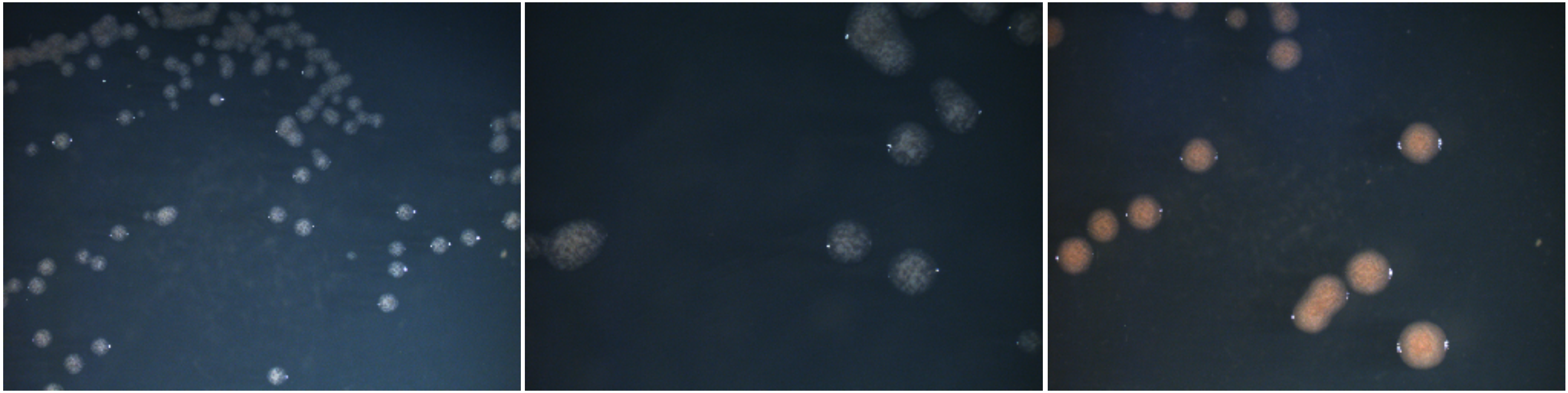

Bottom View

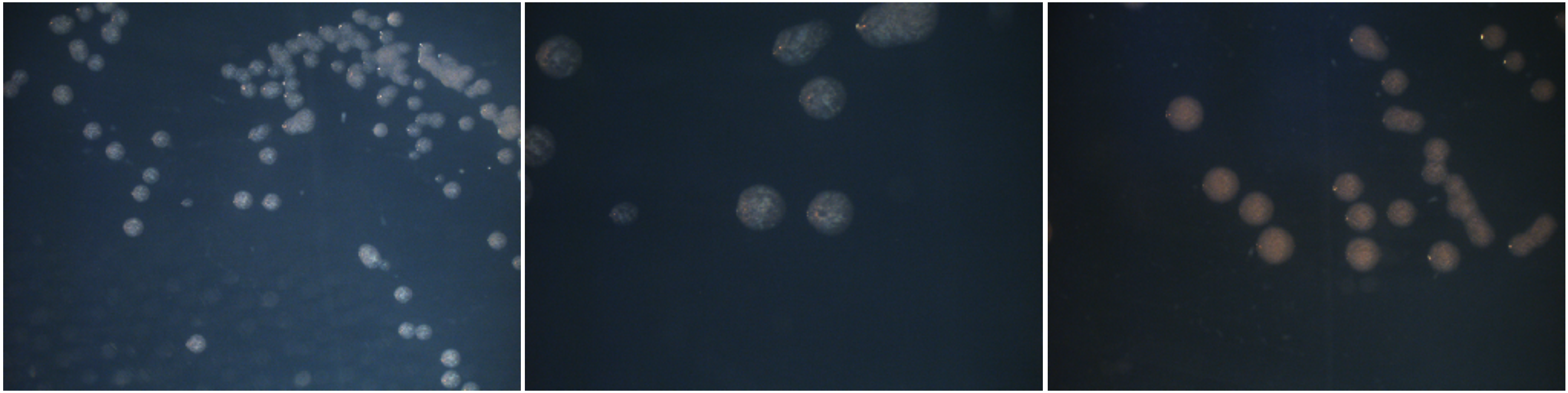

Sphingobacterium, Lab0004

Top View

Day 1		Day 2
0.8x	2.0x	0.8x

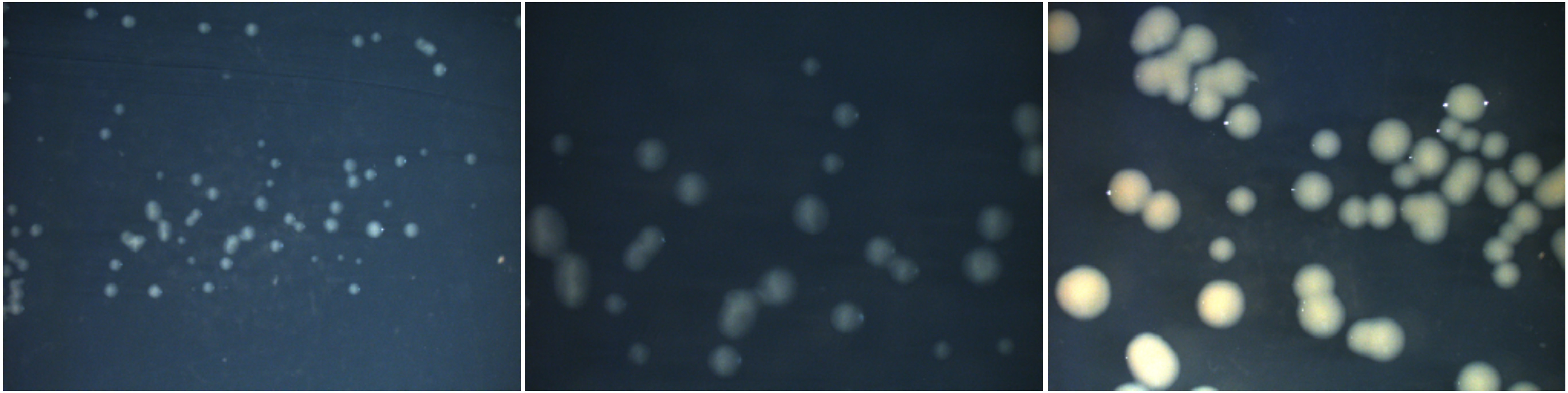

Bottom View

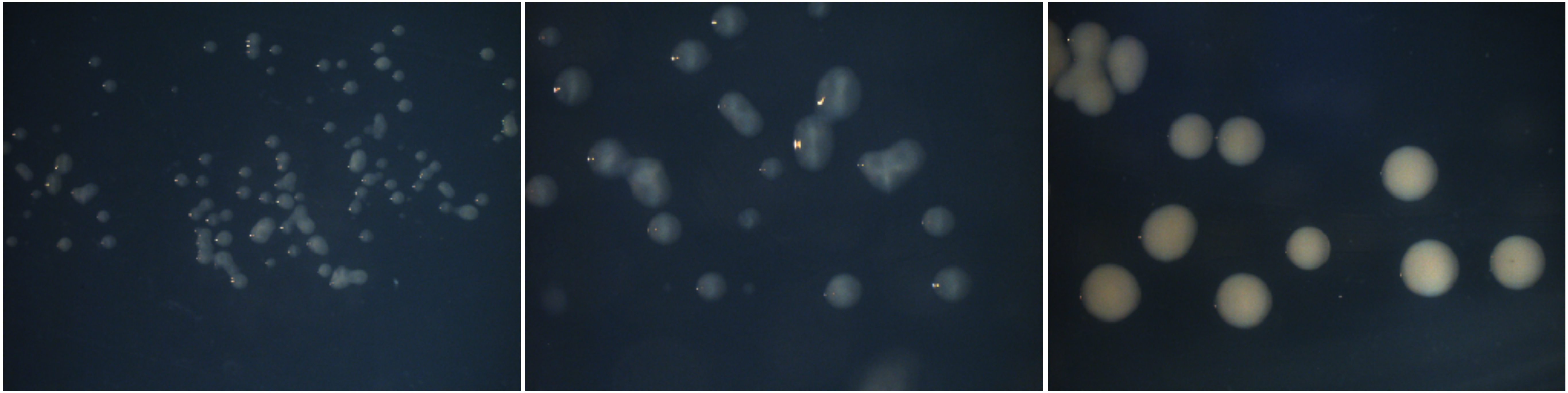

Acinetobacter, River0008

Top View

Day 1 | Day 2

0.8x | 2.0x | 0.8x

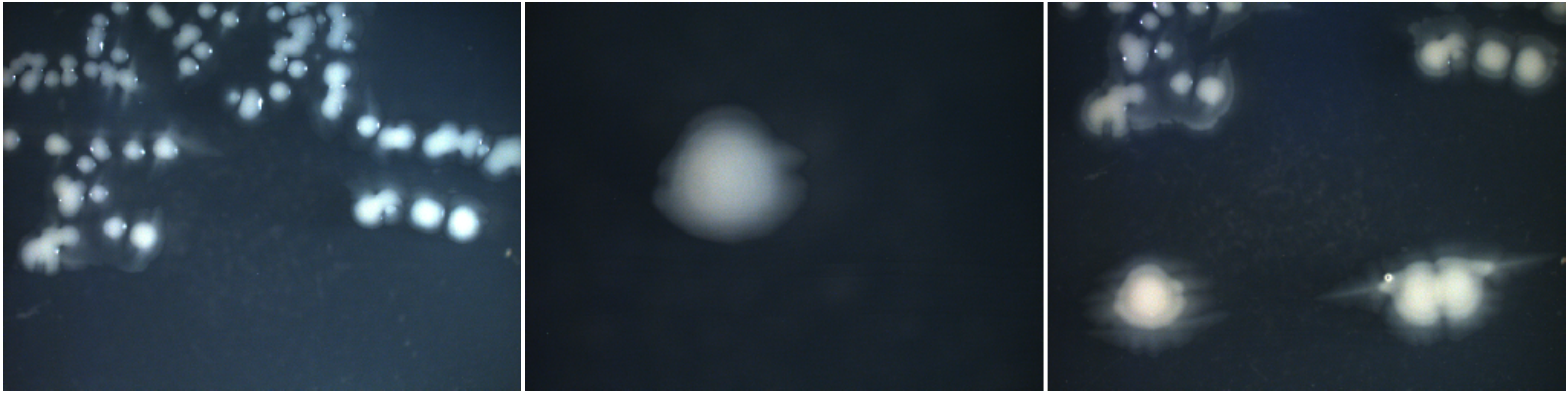

Bottom View

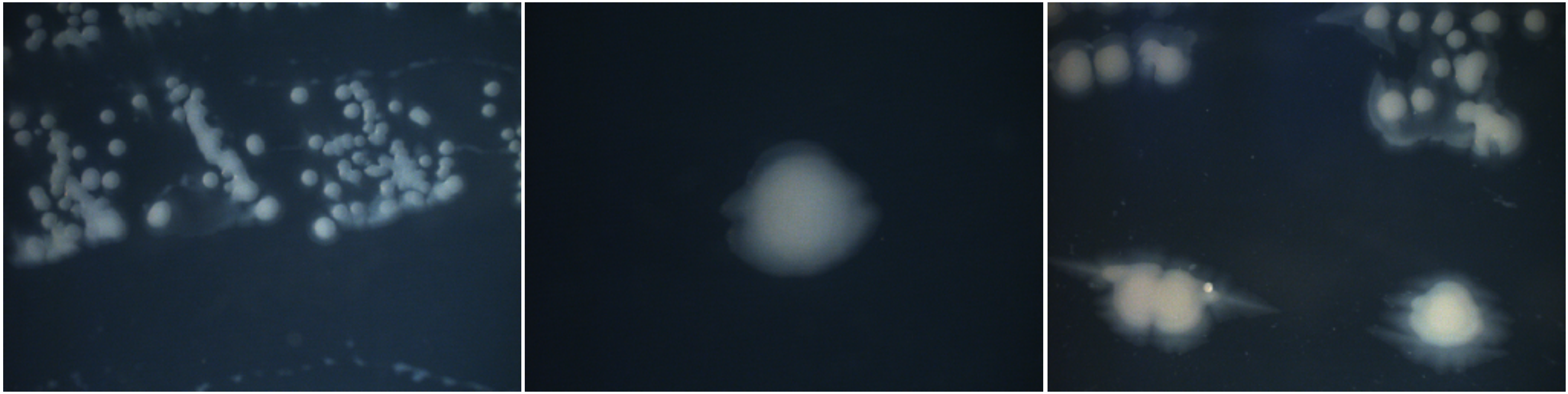

Flectobacillus

Older

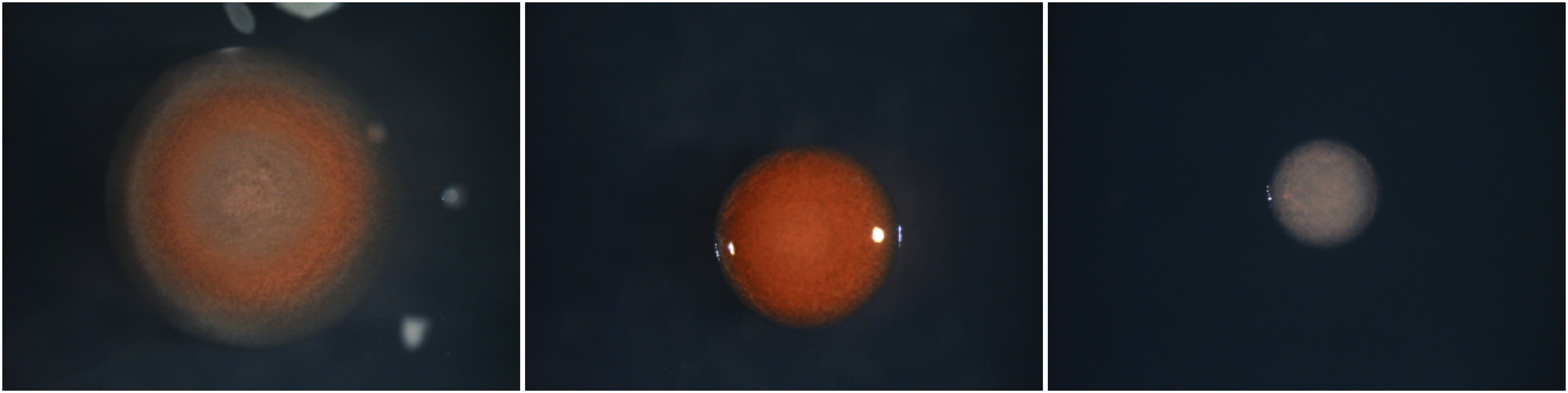

Supplement: Supplementary file 1 [file Presentation_1.PDF]
