# Supplementary material for: Diet–Microbiota Interactions Alter Mosquito Development
Source: Front Microbiol. 2021 Jun 8;12:650743. doi: 10.3389/fmicb.2021.650743 (PMC8217444; doi:10.3389/fmicb.2021.650743)
Supplement: Supplementary file 2 [file Data_Sheet_1.PDF]

Supplementary Figures and Tables.

Diet-microbiota interactions alter mosquito development

Vincent G. Martinson[1, 2]* and Michael R. Strand[1]*
[1] Department of Entomology, University of Georgia, Athens, GA 30602, USA
[2] Department of Biology, University of New Mexico, Albuquerque, NM 87131, USA

Address correspondence to either:
Vincent G. Martinson, vmartinson@unm.edu
Michael R. Strand, mrstrand@uga.edu

This PDF file includes:

Figures S1 to S9
Tables S1 to S4

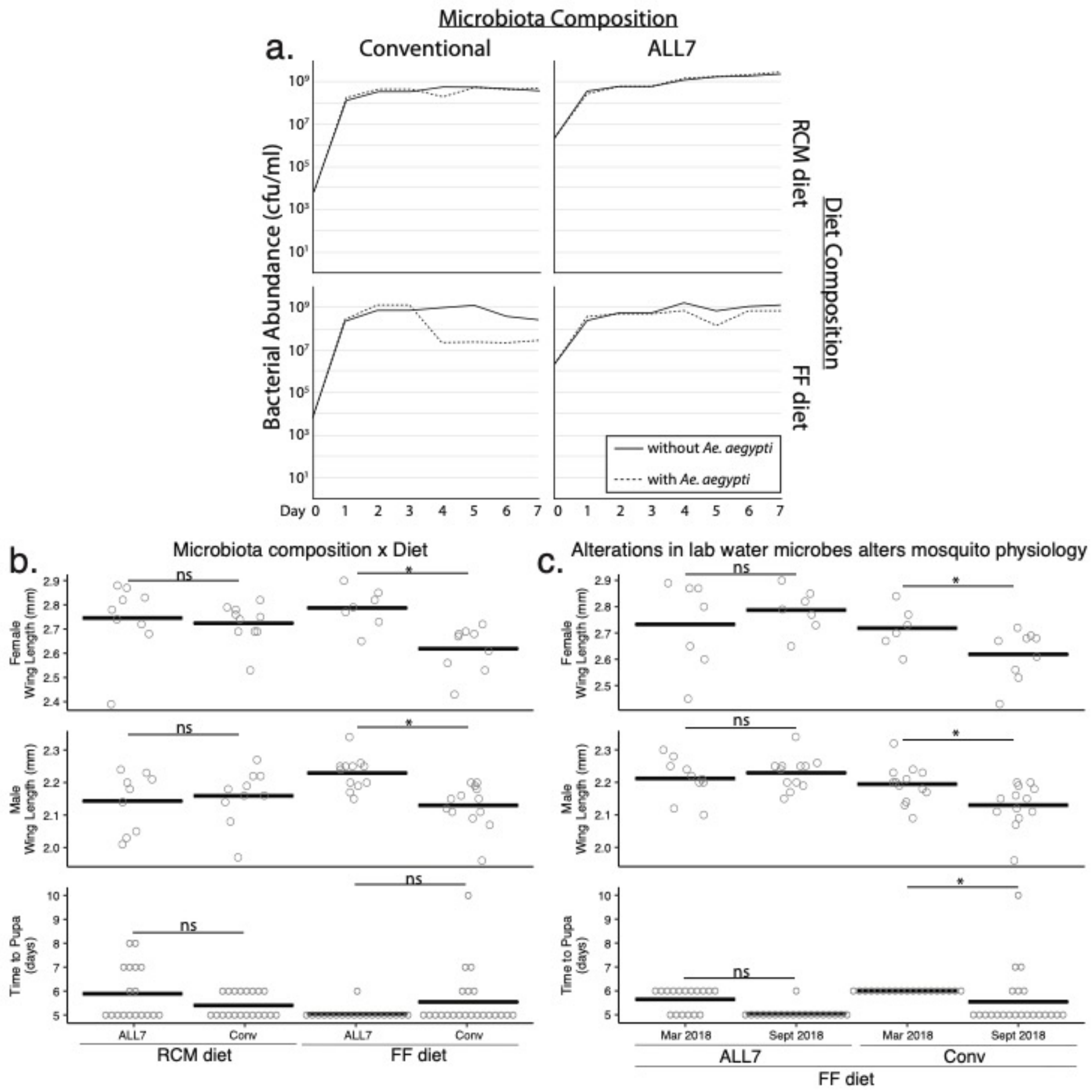

Figure S1. Daily abundance of total colony forming units (cfu) in rearing water (a) and the corresponding *A. aegypti* performance metrics (b). *A. aegypti* reared with the conventional or ALL7 microbiota do not differ in metrics on RCM diet but they are significantly different on the FF diet (b). However, measurements taken six months apart (c) show that *A. aegypti* had fewer differences in developmental performance when reared with the ALL7 microbiota than with the contemporary laboratory rearing water microbial community (Conv) where larvae differed significantly in all three developmental metrics.

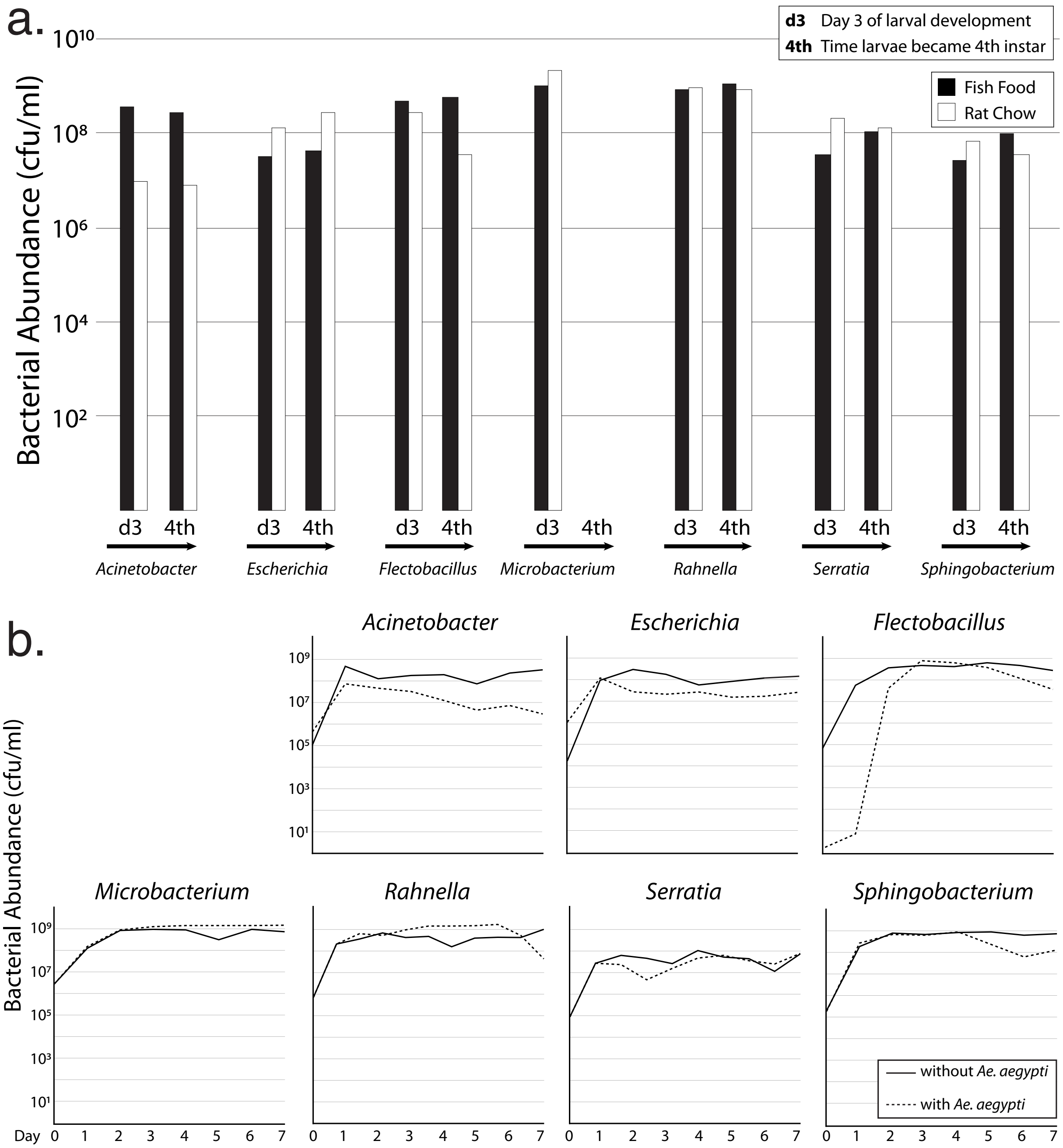

Figure S2. Measurements of bacterial cells present in the rearing water during mosquito development. a) The cfu counts for mono-inoculated bacteria in RCM or FF diet treatments at different time points during mosquito development. b) Daily counts of cfu for individual bacterial species that were grown alone or used to rear *A. aegypti* larvae through development on the FF diet.

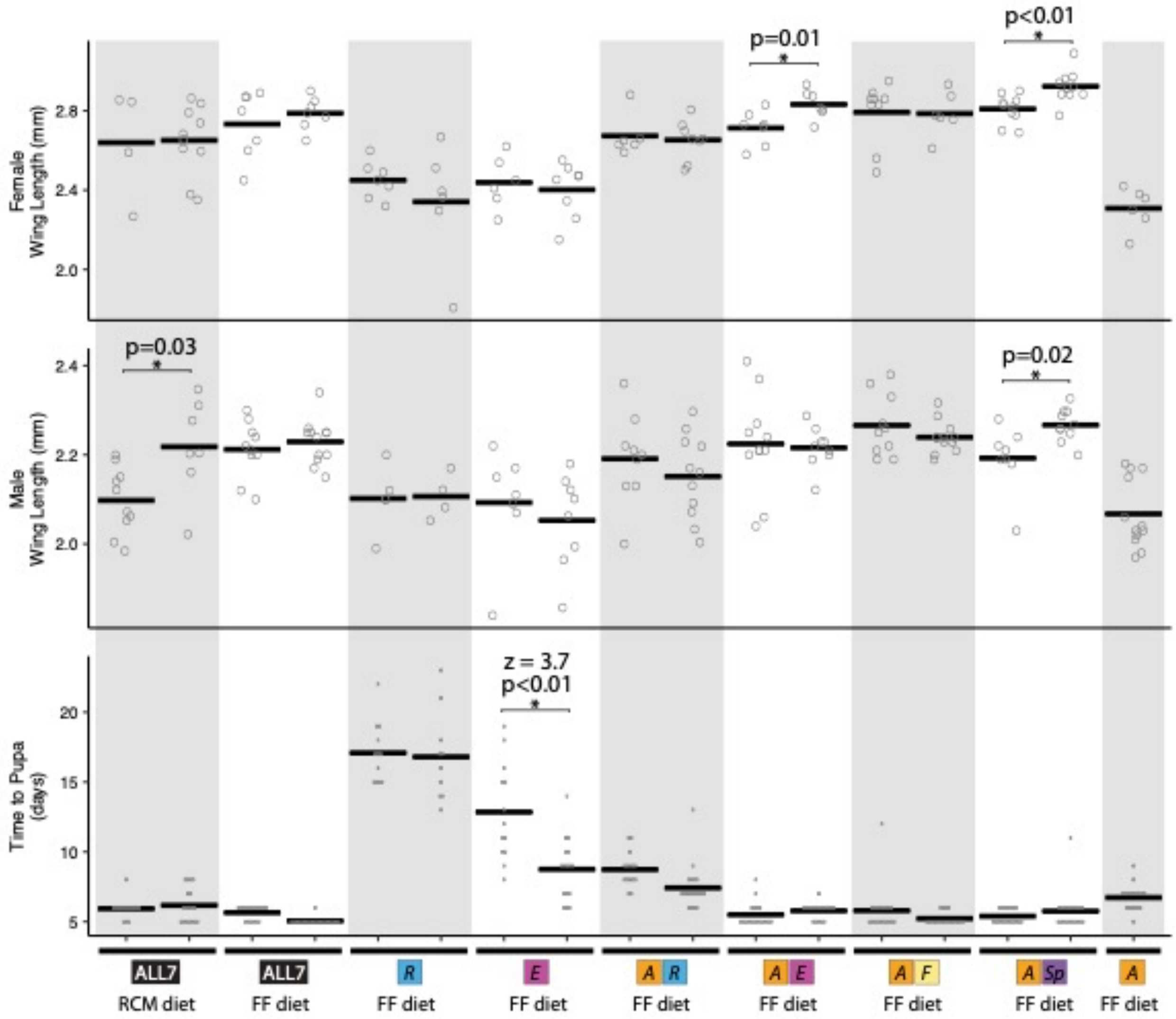

Figure S3. Within-treatment replication across different experimental variables including 1) diet, 2) one-member microbiotas, 3) two-member microbiotas. While some measurements were significantly different between replications, the majority were similar in their overall pattern. Specifically, one-member microbiotas produced smaller individuals, and two-member microbiotas produced larger individuals. *Acinetobacter*, FF diet treatment was included for comparison. Bars indicate mean abundance for the treatment. Asterisks indicate a significant difference [wing length, t-test; pupation time, Wilcoxon test].

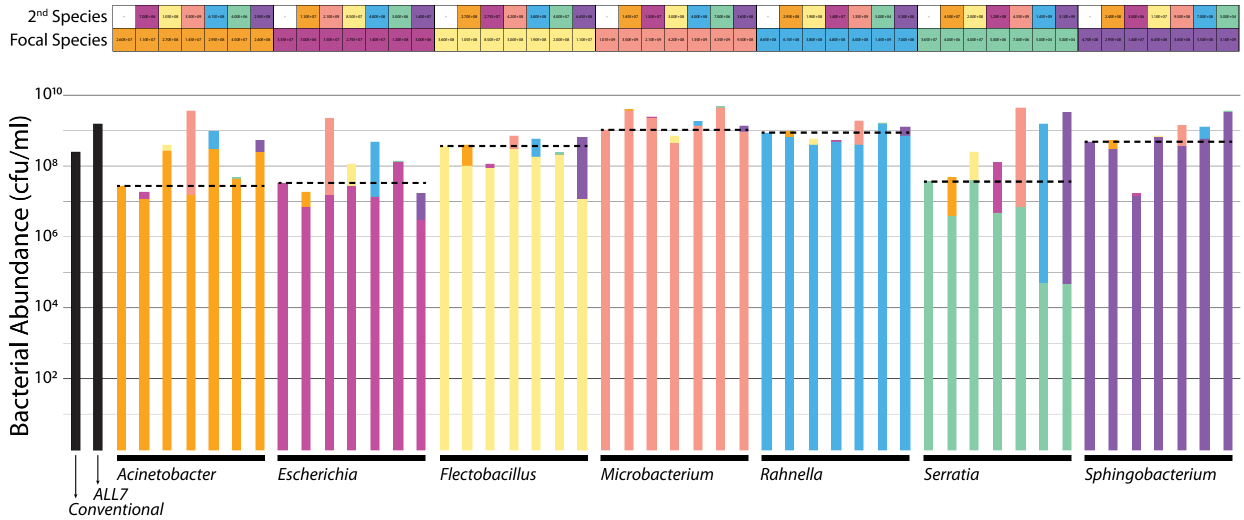

1) Focal species abundance	z = -.25, p = 0.80	z = -0.75, p = 0.45	z = 1.25, p = 0.21	z = -0.25, p = 0.80	z = -0.75, p = 0.45	z = -0.75, p = 0.45	z = 0.00 p = 1.00
2) Total bacterial abundance	z = -0.75, p = 0.45	z = -0.25, p = 0.80	z = -0.25, p = 0.80	z = -0.75, p = 0.45	z = -0.25, p = 0.80	z = -1.25, p = 0.21	z = -0.75, p = 0.45

3) Total bacterial abundance	Conventional ALL7 Monoxenic Pairs	$X^2(3) = 4.53$, p = 0.21

Figure S4. The cfu counts of individual bacterial species in the water column containing developing mosquito larvae, three days post inoculation. Each focal species has cfu counts for monoxenic rearing (first bar) and for all six pairwise combinations with a second bacterium. Focal species color is designated by the monoxenic bar where each dashed line originates. Counts of each focal species in monoxenic conditions were compared to counts in pairwise bacterial sets of 1) the focal species, and 2) the total bacterial abundance (sum of paired species abundances). 3) Total bacterial abundance was compared among conventional, ALL7, monoxenic, and paired communities. Comparisons were made with Wilcoxon and Kruskal-Wallis tests.

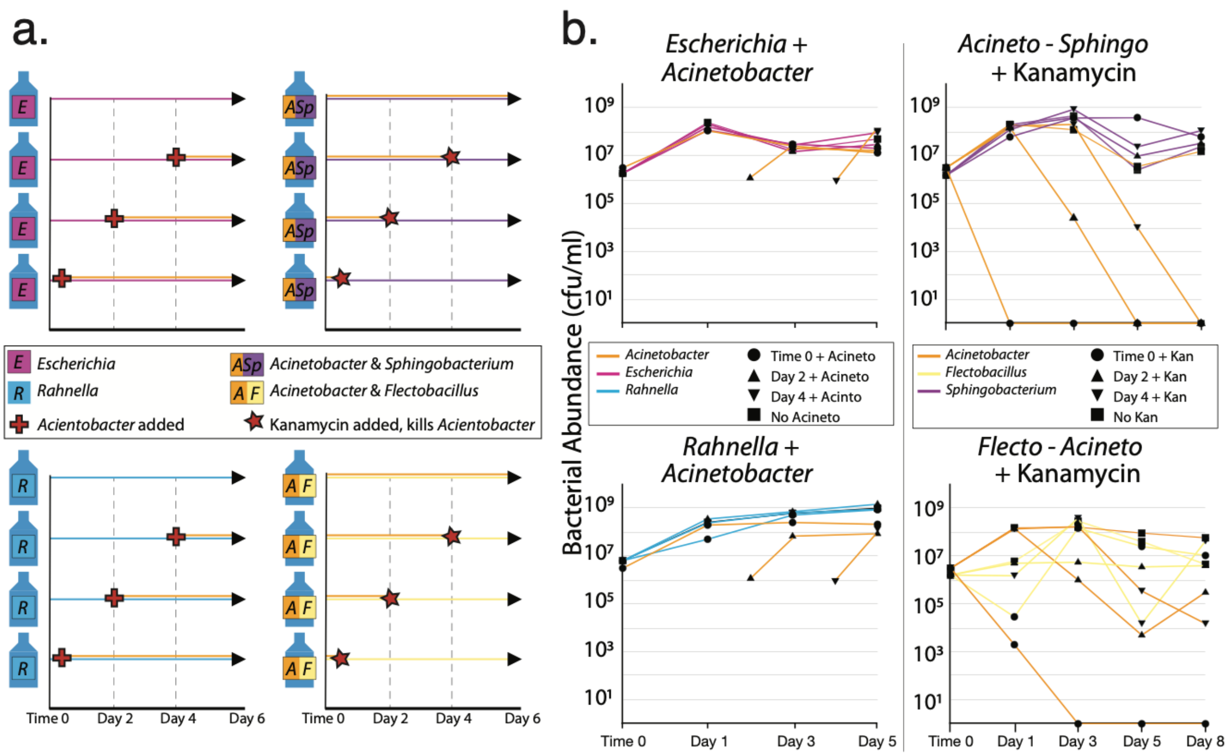

Figure S5. Addition or removal of a second bacterial species during *A. aegypti* larval development a) experimental design to test the effect of pairwise bacterial microbiota, b) counts of cfu for individual bacterial species during the experiment.

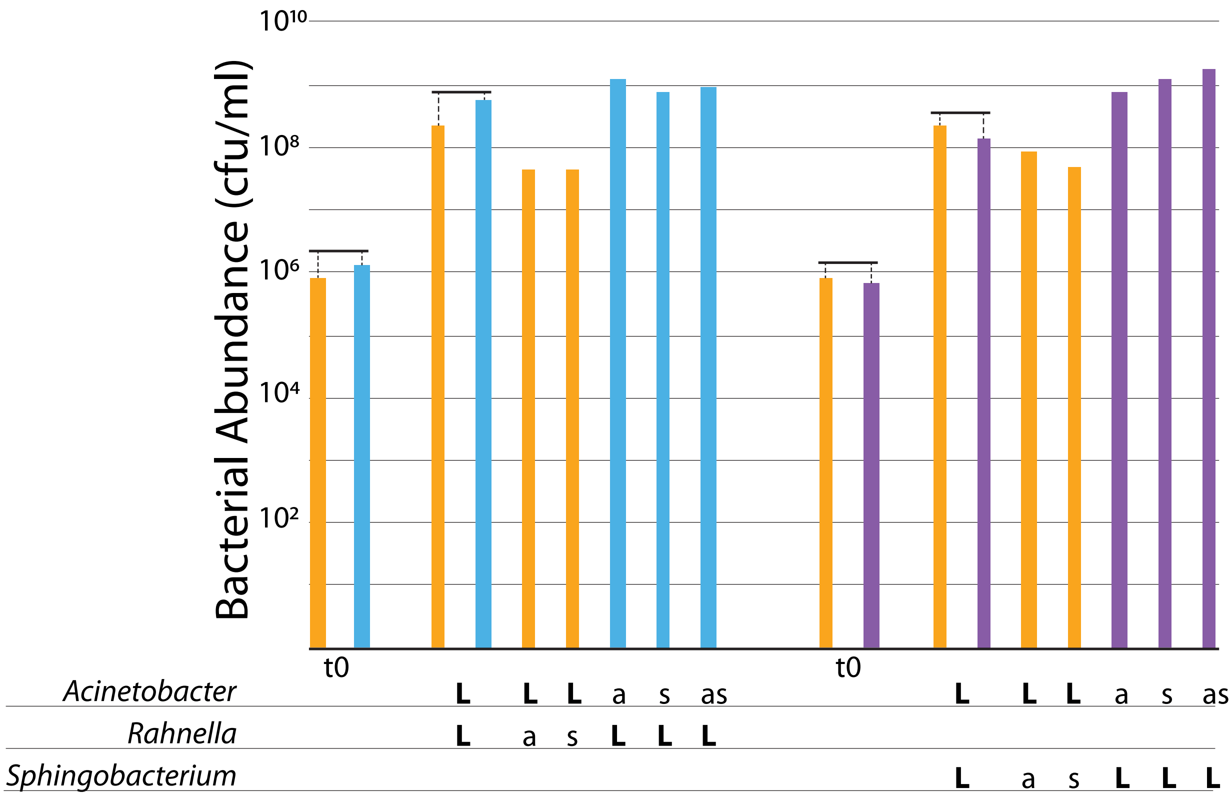

Figure S6. The cfu counts for bacterial species paired in combinations of live and dead cells. All measurements were taken on day three of rearing except those of the initial input at t0. t0, Time 0 (initial input); L, Live bacteria; a, dead cells (autoclaved); s, dead cells (sonicated and 0.2μm filtered). Colors for each bacterium correspond to Figure S3.

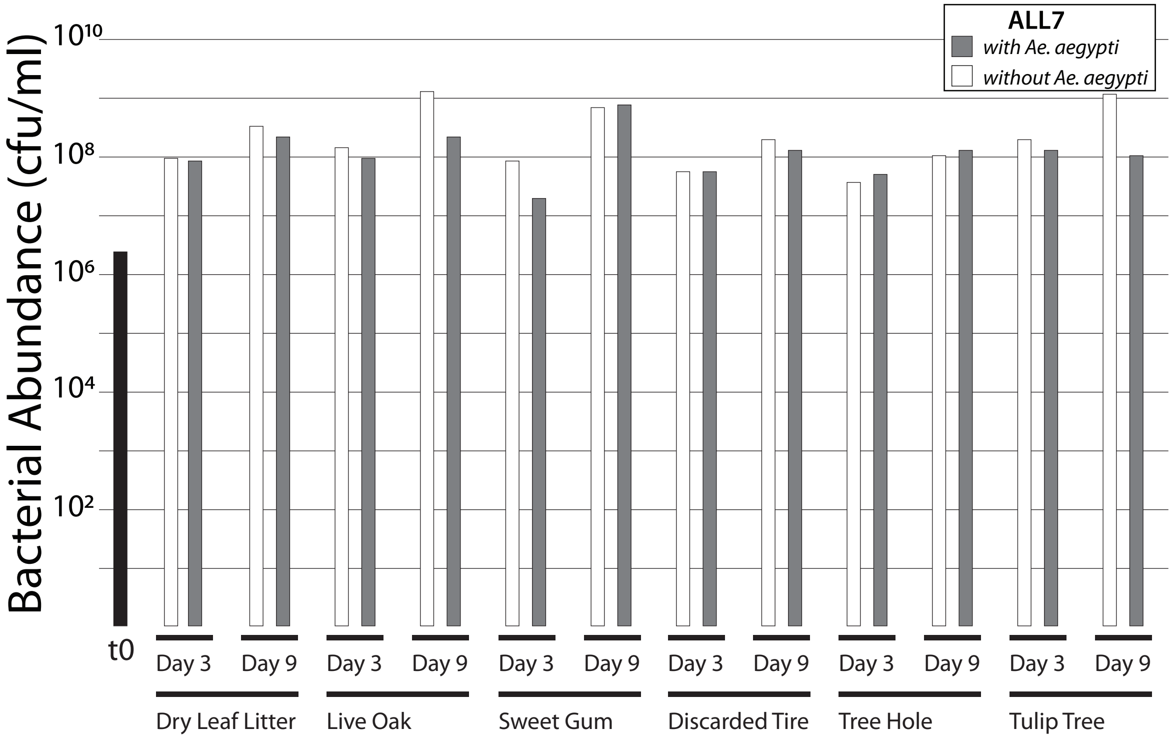

Figure S7. The cfu counts for the ALL7 microbiota grown in different natural diets with and without *A. aegypti* larvae. t0, Time 0 (initial input); Day 3, three days post hatching; Day 9, nine days post hatching. Measurements are for total bacterial cells present in the rearing water.

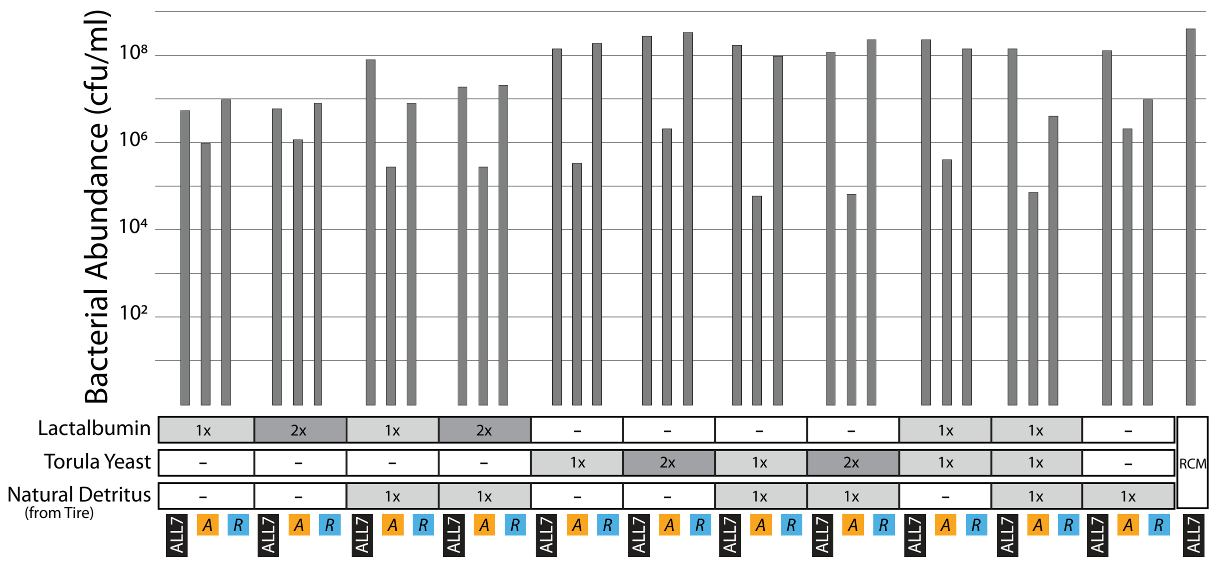

Figure S8. Performance metrics of *A. aegypti* reared on diet combinations of lactalbumin, torula yeast, and natural detritus. For each diet composition, mosquitoes were reared with three microbiotas; ALL7, *Acinetobacter* (A), and *Rahnella* (R). Measurements of bacterial abundance (cfu/ml) in rearing water on the third day post inoculation. 1x, diet provisioned at 1/3 quantity of RCM diet; 2x, diet provisioned at 2/3 quantity of RCM diet; –, omitted from diet.

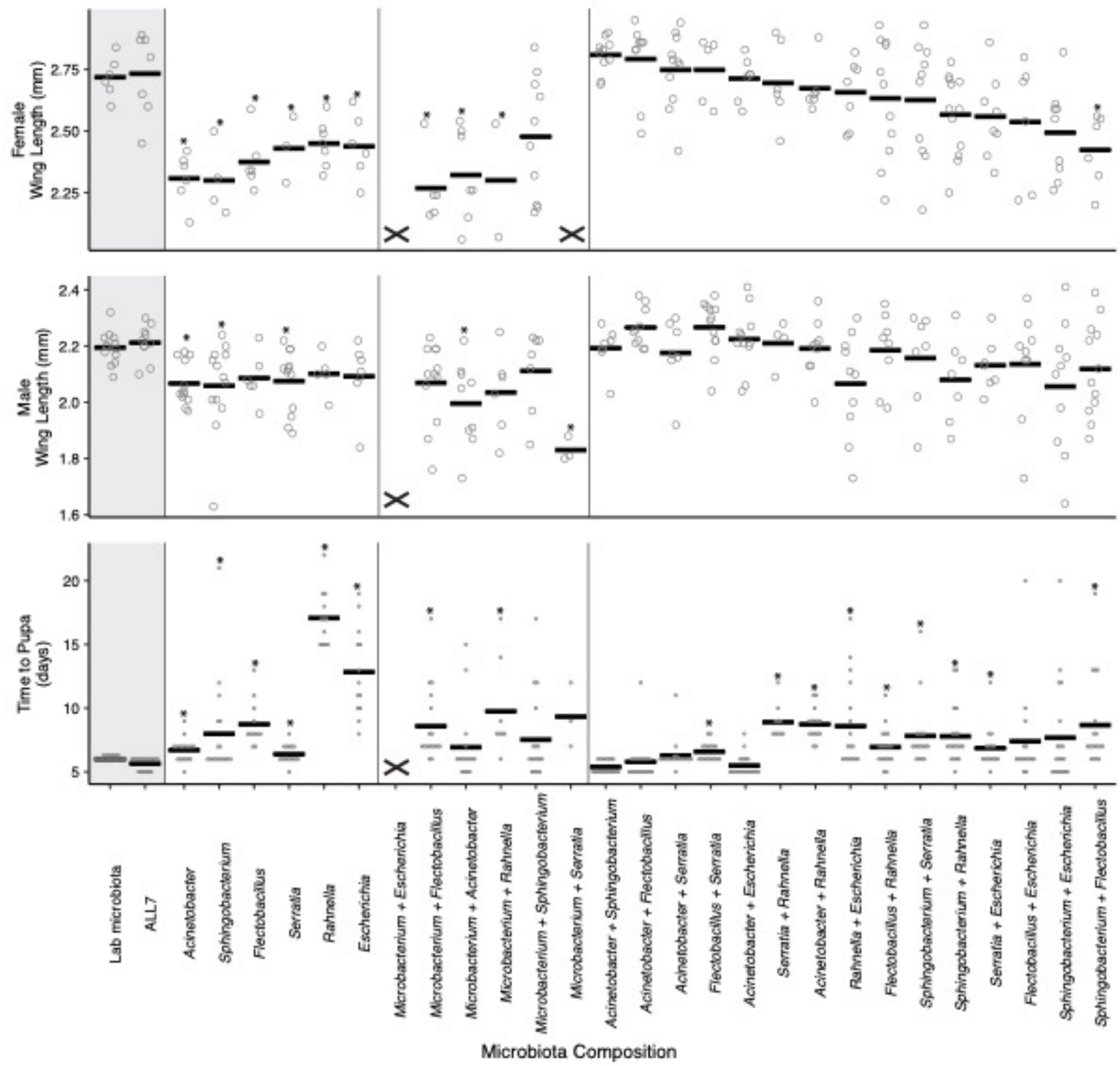

Figure S9. Performance metrics of *A. aegypti* reared with FF diet and different microbiotas: laboratory, ALL7, individual bacteria, and bacterial pairs. This figure allows comparisons between mosquitoes reared monoxenically and those reared with paired microbial communities. The "X" marks at the bottom of data columns for "Time to Pupa" and "Wing Length" indicate that larvae did not develop into pupae or adults, respectively. Bars indicate mean abundance for the treatment. Asterisks indicate a significant difference for treatment relative to the community microbiota control = ALL7 [wing length, Dunnett's ($P < 0.05$); pupation time, Steel's ($P < 0.05$)].

Table S1.

Table S1. Bacterial strains used in this manuscript that composed the ALL7 community.

Genus	Species	Strain	GenBank Accession (16S rRNA / Genome)	Phylum	Class	Order	Family	Collection Source	Ampicillin (100ug/ml)	Tetracycline (10ug/ml)	Kanamycin (50ug/ml)	Chloramphenicol (25ug/ml)	Spectinomycin (50ug/ml)
Microbacterium		Lab0001	MN544615	Actinobacteria	Actinobacteria	Actinomycetales	Microbacteriaceae	Laboratory Mosquitoes	no	no	resistant	no	resistant
Sphingobacterium		Lab0004	MN544616	Bacteroidetes	Sphingobacteria	Sphingobacteriales	Sphingobacteriaceae	Laboratory Mosquitoes	no	no	resistant	no	resistant
Flectobacillus		Rain0001	MN544619	Bacteroidetes	Cytophagia	Cytophagales	Cytophagaceae	Wild 1	no	no	resistant	no	resistant
Rahnella		Rain0009	MN544617	Proteobacteria	Gammaproteobacteria	Enterobacteriales	Enterobacteriacae	Wild 1	no	no	no	no	no
Serratia		Rain0010	MN544614	Proteobacteria	Gammaproteobacteria	Enterobacteriales	Enterobacteriacae	Wild 1	no	no	no	no	no
Acinetobacter		River0008	MN544618	Proteobacteria	Gammaproteobacteria	Pseudomonadales	Moraxellaceae	Wild 2	no	no	no	no	no
Escherichia	*coli*	K-12 substr. MG1655	U00096	Proteobacteria	Gammaproteobacteria	Enterobacteriales	Enterobacteriacae	Lab Strain	no	no	no	no	no

Genus	Species	Strain	Colony Identification		
			24hr growth	48hr growth	>48hr growth
Microbacterium		Lab0001	pinpoint	small, round margin, yellow	medium, round margin, bright yellow
Sphingobacterium		Lab0004	small, round margin, yellow	large, round margin, pale yellow	
Flectobacillus		Rain0001	small, round margin, mottled pale pink	medium, round margin, mottled bright pink	
Rahnella		Rain0009	small, round margin, coarsely mottled white	medium, round margin, coarsely mottled white	
Serratia		Rain0010	pinpoint	small, undulate margin, finely mottled white	
Acinetobacter		River0008	medium, transparent lobate margin, cream center	large, transparent lobate margin, cream center	
Escherichia	*coli*	K-12 substr. MG1655	medium, undulate margin, white	large, undulate margin, white	

Table S2.

Table S2. Macronutrient composition of RCM and FF diets.

		RCM diet components			Laboratory diets	
		LabDiet 5012 (Rat Chow)	Lactalbumin	Torula yeast	RCM diet*	FF diet (TetraColor Trop. Granules)
Macronutrient (% content w/w)	Protein	22	100	50	57.3	47.5
	Fat	4	0	2	2	6.5
	Fiber	5	0	0	1.7	2
Ingredients		ground corn	lactalbumin	torula yeast		fish meal
		dehulled soybean meal				dehulled soybean meal
		fish meal				wheat germ meal
		wheat middlings				wheat flour
		cane molasses				corn gluten
		dehydrated alfalfa meal				feeding oat meal
		soybean oil				potato protein
		ground oats				shrimp meal
		dried beet pulp				dried yeast
		wheat germ				wheat gluten
		brewers dried yeast				monobasic calcium phosphate
		dicalcium phosphate				L-lysine monohydrochloride
		calcium carbonate				lecithin
		salt				algae meal
		DL-methionine				soybean oil
		choline chloride				yeast extract
		cholecalciferol				ascorbic acid (sourse of vitamin C)
		menadione dimethylpyrimidinol bisulfite (source of vitamin K)				cholin choride
		pyridoxine hydrochloride				D-calcium pantothenate
		vitamin A acetate				thiamine mononitrate
		biotin				pyridoxine hydrocholoride
		thiamine mononitrate				bisulfite complex
		vitamin B12 supplement				folic acid
		dl-alpha tocopheryl acetate (form of vitaminE)				biotin
		nicotinic acid				vitamin B12 supplement
		calcium pantothenate				cholecalciferol (source of vitamin D3)
		riboflavin supplement				manganese sulfate
		manganous oxide				zinc sulfate
		zinc oxide				ferrous sulfate
		ferrous carbonate				cobalt acetate
		coppersulfate				beta-carotene
		zinc sulfate				annato extract
		calcium iodate				red 3
		cobalt carbonate				ethoxyquin
						citric acid

* RCM is equal parts LabDiet 5012, Lactalbumin, and Torula Yeast

Table S3.

Table S3. Sample source and tissues/materials of field-collected detritus diets used in this study with pupation and eclosion rates for *A. aegypti* reared with the ALL7 or endemic microbiotas.

Common Name	Species name	Material Irradiated	ALL7 microbiota		Endemic microbiota	
			Pupation	Adults	Pupation	Adults
Tulip Tree	*Liriodendron tulipifera*	Fresh leaves	5/20	4/20	–	–
Sweet Gum	*Liquidambar styraciflua*	Fresh leaves	0/20	0/20	–	–
Live Oak	*Quercus virginiana*	Fresh leaves	0/20	0/20	–	–
Dry Leaf Litter	Mixed Species	Dried leaves	0/20	0/20	–	–
Tree Hole	Inside *L. styraciflua*	Wet detritus, mixed leaves, sticks, dirt	0/20	0/20	2/20	2/20
Discarded Tire	Inside a car tire	Wet detritus, mixed leaves, sticks, dirt	0/20	0/20	1/20	1/20

Table S4.

Table S4. Developmental measurements for adult *A. aegypti* reared with detritus diets.

Diet	Microbiota	Sex	Wing Length (mm)	Time to pupa (days)
Tulip Tree	All7	Female	1.95	19
Tulip Tree	All7	Male	2.36	21
Tulip Tree	All7	Male	2.18	25
Tulip Tree	All7	Male	2.24	21
Tulip Tree	All7	Died as pupa	–	16
TreeHole	Endemic Microbiota	Male	1.64	22
TreeHole	Endemic Microbiota	Male	1.61	24
Discarded Tire	Endemic Microbiota	Male	1.73	11
